# Clinical and *in vitro* models identify distinct adaptations enhancing *Staphylococcus aureus* pathogenesis in human macrophages

**DOI:** 10.1371/journal.ppat.1012394

**Published:** 2024-07-11

**Authors:** Dustin R. Long, Elizabeth A. Holmes, Hsin-Yu Lo, Kelsi Penewit, Jared Almazan, Taylor Hodgson, Nova F. Berger, Zoe H. Bishop, Janessa D. Lewis, Adam Waalkes, Daniel J. Wolter, Stephen J. Salipante

**Affiliations:** 1 Division of Critical Care Medicine, Department of Anesthesiology and Pain Medicine, University of Washington School of Medicine, Seattle, Washington, United States of America; 2 Department of Laboratory Medicine and Pathology, University of Washington School of Medicine, Seattle, Washington, United States of America; 3 Department of Pediatrics, University of Washington School of Medicine, Seattle, Washington, United States of America; Columbia University, UNITED STATES OF AMERICA

## Abstract

*Staphylococcus aureus* is a facultative intracellular pathogen of human macrophages, which facilitates chronic infection. The genotypes, pathways, and mutations influencing that phenotype remain incompletely explored. Here, we used two distinct strategies to ascertain *S*. *aureus* gene mutations affecting pathogenesis in macrophages. First, we analyzed isolates collected serially from chronic cystic fibrosis (CF) respiratory infections. We found that *S*. *aureus* strains evolved greater macrophage invasion capacity during chronic human infection. Bacterial genome-wide association studies (GWAS) identified 127 candidate genes for which mutation was significantly associated with macrophage pathogenesis *in vivo*. In parallel, we passaged laboratory *S*. *aureus* strains *in vitro* to select for increased infection of human THP-1 derived macrophages, which identified 15 candidate genes by whole-genome sequencing. Functional validation of candidate genes using isogenic transposon mutant knockouts and CRISPR interference (CRISPRi) knockdowns confirmed virulence contributions from 37 of 39 tested genes (95%) implicated by *in vivo* studies and 7 of 10 genes (70%) ascertained from *in vitro* selection, with one gene in common to the two strategies. Validated genes included 17 known virulence factors (39%) and 27 newly identified by our study (61%), some encoding functions not previously associated with macrophage pathogenesis. Most genes (80%) positively impacted macrophage invasion when disrupted, consistent with the phenotype readily arising from loss-of-function mutations *in vivo*. This work reveals genes and mechanisms that contribute to *S*. *aureus* infection of macrophages, highlights differences in mutations underlying convergent phenotypes arising from *in vivo* and *in vitro* systems, and supports the relevance of *S*. *aureus* macrophage pathogenesis during chronic respiratory infection in CF. Additional studies will be needed to illuminate the exact mechanisms by which implicated mutations affect their phenotypes.

## Author summary

*Staphylococcus aureus* is a significant cause of disease and can cause chronic infections that are extremely difficult to treat. One of the reasons that *S*. *aureus* persists so effectively in the human body is its ability to enter and survive within macrophages, which protects the bacteria from immune clearance and antibiotic therapies. A better understanding of the genes and mechanisms that *S*. *aureus* uses to infect macrophages could lead to new, more effective treatments for chronic infection. Here, we identified gene mutations associated with improved macrophage pathogenesis in *S*. *aureus* by studying isolates from chronic respiratory infection *in vivo* and using a laboratory macrophage model *in vitro*. We identified multiple, previously unknown genes and functions that can influence *S*. *aureus* macrophage pathogenesis.

## Introduction

*Staphylococcus aureus* is an important cause of chronic disease affecting a variety of organ systems, and incurs significant morbidity and mortality worldwide [1]. Chronic *S*. *aureus* infections can persist or relapse for years to decades, even despite therapeutic intervention with antibiotics [2–7]. The tenacity of *S*. *aureus* during chronic infection is attributable in part to the emergence of adaptations that increase the organism’s fitness over time [8–11]. Among other features, studies have shown that changes promoting the invasion and persistence of *S*. *aureus* within host cells, particularly macrophages [1,12–18], occur frequently in chronic diseases [16,19–23]. As a facultative intracellular pathogen, *S*. *aureus* is able to evade the immune system and establish a microbial reservoir able to perpetuate persistent infection in a variety of clinical conditions [1,12–15,17,18,21,24,25].

Factors and pathways important for intracellular invasion and survival of *S*. *aureus* have been identified in prior studies [12–14,26]. These comprise a diverse range of mechanisms affecting intracellular survival and subsequent dispersal, including manipulation of host cell autophagy, metabolism, programmed cell death, and self-modulation of virulence gene expression [27]. Nevertheless, the genes contributing to such phenotypes remain incompletely characterized, owing to the inherent complexity of these processes and their dependence on both strain- and host-encoded features [26]. Moreover, prior studies have focused on identifying genes that are essential to *S*. *aureus* intracellular pathogenesis, rather than spontaneous chromosomal mutations that are selected during infection and that drive such phenotypes *in vivo*.

Here, we employed a multiphasic approach to investigate mutations affecting intracellular pathogenesis of *S*. *aureus* within human macrophages, using cystic fibrosis (CF) as a paradigm for chronic infection [1,18,21]. In order to explore pathogenesis-enhancing mutations arising *in vivo* during human infection, we conducted genome-wide association studies (GWAS) of clonally related *S*. *aureus* isolates longitudinally obtained from CF patient airways at the time of initial isolation and late in the course of disease [4]. To identify mutations relevant to those same phenotypes in defined genetic backgrounds and under well-controlled experimental conditions, we separately performed artificial selection of two phylogenomically distinct *S*. *aureus* laboratory strains for intracellular pathogenesis using a scalable human macrophage cell line model (THP-1) [28,29]. Using a combination of isogenic transposon mutant knockouts [30] and a newly developed CRISPR interference (CRISPRi) [31] knockdown vector, we subsequently performed functional validation of genes affecting *S*. *aureus* macrophage pathogenesis identified by both strategies.

## Materials and methods

### Laboratory *S*. *aureus* strains, cell lines, and growth conditions

*S*. *aureus* strain ATCC29213 was obtained from the American Type Culture Collection. Strain JE2 and JE2 transposon mutants were from the Biodefense and Emerging Infections Research Resources Repository (BEI Resources). All bacteria in this study were maintained in LB medium supplemented with hemin, menadione, and thymidine (sup-LB) in order to support potentially relevant auxotrophic mutants [32].

THP-1 cells were obtained from ATCC and cultured according to the supplier’s recommendations at 37°C in a humidified 5% (v/v) CO2 air atmosphere in RPMI 1640 medium (Gibco) supplemented with 0.1 mg/ml l-glutamine, 0.1 mg/ml streptomycin, 100 U/ml penicillin (PAA) and 20% (v/v) Nu-Serum Serum Replacement (Corning).

### *S*. *aureus* clinical isolates

Control clinical isolates of methicillin-sensitive *S*. *aureus* (MSSA) and methicillin-resistant *S*. *aureus* (MRSA), derived from individuals without CF and from sites not involved in active disease, were obtained from fully de-identified surveillance swabs [33] at the University of Washington Clinical Microbiology Laboratory following the completion of clinical testing. MSSA isolates were obtained by streaking swabs onto mannitol-salt agar, and MRSA isolates from streaking onto chromogenic MRSA selective plates (bioMerieux, Marcy-l’Étoile, France). Control clinical isolates (n = 180) were subjected to whole-genome sequencing as described below.

Clinical isolates from children with CF were previously described and characterized by whole-genome sequencing [4], constituting 1,382 longitudinally-collected *S*. *aureus* isolates collected from 246 participants. For clonally related isolates derived from the same patient (n = 237 lineages), we selected the first *S*. *aureus* isolate that was initially cultured from the patient and the last isolate temporally collected during the study period [4]. Secondary data, but no human subjects, were analyzed in this study. From this collection of paired clinical isolates, we further reduced the set analyzed in this study to a cohort of 57 lineages identified from the phylogeny of that collection [4] which spanned the genomic diversity present within the collection.

For each such lineage, we identified a matching, phylogenomically similar control strain to minimize lineage-specific effects [34] in comparisons between clinical and control strains. To accomplish this, whole-genome sequencing data from CF and control isolates were subjected to *de novo* assembly using ABySS 2.0 [35]. We generated a core-genome alignment containing the entire control population and the first isolate from each CF lineage using ROARY [36]. SNP-sites [37] was then used to calculate pairwise core-genome distances between CF-derived and control isolates. For each CF-derived isolate, a phylogenomically similar match from the control population was then identified using an optimal matching strategy to minimize the global distance between all matched cases and controls in the set [38] with a maximum core-genome SNP caliper of 6% and a maximum control to clinical isolate matching ratio of two. This process resulted in matching of the 57 CF-derived isolate pairs to 33 distinct control isolates. All isolates (57 CF first-collected isolates, 57 CF last-collected isolates, and 33 matched control isolates) were subjected to characterization by intracellular pathogenesis assays as described above (**S1 Table**).

### Intracellular pathogenesis assays and selection procedures

48 hours prior to bacterial infection, THP-1 cells were differentiated into monocytes with PMA (Sigma) solubilized in DMSO at a final concentration of 10 ng/ml [39–41]. PMA-containing medium was removed from cells 24 hours after treatment. Cells were washed with incubation medium (IM, RPMI 1640 containing no amendments) and incubated for 24 additional hours.

Bacteria were expanded overnight in sup-LB broth, washed twice in Dulbecco’s phosphate-buffered saline (DPBS) (Difco) to remove soluble toxins, and cell density assessed by optical density at 600 nm (OD600). Six replicates were independently passaged in parallel for each strain.

Intracellular pathogenesis assays were based on previously described lysostaphin protection assays [42]. Briefly, bacteria were combined with differentiated THP-1 cells at a multiplicity of infection (MOI) of 10 in IM for 1 h, with aliquots of inoculum plated to empirically determine the number of bacteria applied. The medium was replaced with RPMI 1640 containing 50 μg/ml lysostaphin (Sigma), and selections were performed at 37°C with 5% atmospheric CO2. 3 h or 48 h post-inoculation, host cells were washed with DPBS and lysed with water. Serial dilutions of lysate were plated onto sup-LB agar to evaluate the number of viable bacteria. At least 4 independent replicates were used to assess the number of viable cells at both 3 and 48 hours. Viable bacteria counted at 3 h were normalized to empiric counts of the bacteria initially applied to determine the proportion of inoculated cells remaining viable after early macrophage entry (measurement of “macrophage invasion”). Viable organisms counted at 48 h were normalized to the number of viable organisms counted at 3 hours for the same isolate (“macrophage survival index”). Measurements of invasion phenotypes were normalized to in-run controls of the parental JE2 or ATCC29213 strains, as appropriate, to control for possible batch effects, whereas the macrophage survival index was inherently normalized for invasion efficiency. Invasion testing of clinical isolates and lineage-matched controls were normalized to an in-run JE2 control. Parental JE2 and ATCC29213 strain invasion was measured as absolute (non-normalized) values collected in a single experiment, due to lack of an external comparator.

For passaging experiments, procedures were completed essentially as described for intracellular pathogenesis assays, except that an initial MOI of 100 was used, lysate was collected only at 48 h, and bacteria were transferred to sup-LB broth for overnight expansion prior to the next passage. As the bacteria became more efficient at infection with increased passaging, it was necessary to empirically reduce the MOI over time to prevent host cells from undergoing lysis during infection while maintaining selective pressure for mutants with increased cytotoxic capability. Consequently, for the first 8 passages, an MOI of 100 was used, after which the MOI was reduced to 10.

### Whole-genome sequencing and variant analysis

Whole-genome sequencing was performed as previously described [43] using MiSeq and NextSeq500 platforms with 300 cycle chemistries (Illumina, San Diego, CA). Briefly, variant calling was conducted as before [43] utilizing reference genomes identified as those most closely matching the *de novo* assemblies of sequenced isolates. Mutations were manually verified using the Integrative Genomics Viewer [44] prior to annotation of sequence features. Common gene names were established using BLAST [45] searches of target gene sequences against *S*. *aureus* reference genomes and by cross-correlating gene identifiers against pan-genome identifiers in the AureoWiki database [46]. MLST was determined using mlst-2.0.3 (https://github.com/tseemann/mlst) in conjunction with the PubMLST database [47].

### Bacterial genome-wide association studies

Three complementary classes of bacterial GWAS were conducted using clinical isolates and matched control strains to identify associations between bacterial genotypes and macrophage pathogenesis phenotype measurements.

To assess the association of specific genotypes with phenotypes in this phylogenetically diverse collection, we performed reference-free unitig analysis with pyseer (v1.3.10) [48]. We adjusted for population structure using a mixed effects model with an approximate maximum likelihood phylogeny constructed from the ROARY core-genome alignment using Fastree2.1.11 [49]. Phenotype measurements for macrophage invasion and macrophage survival index were provided as continuous variables. Both CF isolates and matched controls were included in this analysis.

Different mutations occurring in a gene may confer equivalent phenotypic effects encoded by distinct genotypes. Unitig-based analysis has less power to detect this class of genotype-phenotype association, which is better assessed using kernel-based models of association between the collective “burden” of mutations within a gene and a phenotype. We therefore performed burden testing using the pyseer rare variants method, considering all non-synonymous mutations as equally weighted and using a mixed effects model incorporating the same phylogenetic tree as above. Both CF isolates and matched controls were included in this analysis.

To identify *de novo* mutations occurring within clonally related *S*. *aureus* lineages from an individual host, whole-genome sequences of the first- and last-collected clinical isolate from each patient were compared. For each gene in the pangenome, a multivariable linear regression analysis was performed in R version 4.2.2 to assess the independent association between the presence of a non-synonymous *de novo* mutation in that gene (primary predictor variable) with the absolute value of change in measured phenotype between first and last isolates (outcome variable), with adjustment for changes in two other phenotypes (covariates): biofilm formation (measured as previously [50], **S1 Table**) and the alternate macrophage invasion phenotype measured in this study (i.e., intracellular survival for analysis for invasion activity, and *vice versa*). Only CF isolates were included in this analysis.

Candidate genes implicated in invasion and survival phenotypes *in vivo* by GWAS were selected for functional testing by prioritizing those with high levels of association in a single GWAS model and those that were independently identified in more than one model, including associations with lower individual levels of significance. For unitig analyses, we selected genes having three or more component unitigs achieving Bonferroni-adjusted levels of significance with alpha values of 0.05. For burden testing analyses of both phenotypes, all genes achieving Bonferroni-adjusted significance using an alpha value of 0.05 were selected. For analysis of *de novo* mutation within clinical isolate lineages, we selected all genes with p-values<0.05 and association scores in the top 5%. Lastly, candidate genes showing overlap between GWAS analyses for a phenotype (those with a one or two unitigs meeting Bonferroni-adjusted significance and further identified as undergoing *de novo* mutation between a first-last pair) were selected for validation.

### *S*. *aureus* CRISPRi knockdown

We generated a CRISPRi [31] vector for use in *S*. *aureus* by modifying our earlier system for CAS9-mediated counterselection [51]. Briefly, dCAS9 from pdCAS9 [52] was cloned under expression of the constitutive p23 promoter into plasmid pCN50wt [53] as described previously [51], followed by addition of a synthetic sgRNA under control of the PRAB17 promoter as previously [51]. To facilitate screening for successful guide RNA cloning, a GFP expression cassette was ultimately cloned into the BSAI site of the sgRNA to generate plasmid pCRISPRi (**S2 Table**). Guide RNAs were designed against coding sequences using CRISPOR [54] with screening for off-target effects performed against the *S*. *aureus* USA300_FPR3757 genome, and selecting guide RNAs designed in the “reverse” orientation of the chosen coding sequence. Guide RNA templates were synthesized as ssDNA oligonucleotides (IDT, **S2 Table**) with appropriate tail sequences, then phosphorylated, annealed, and cloned into the BSAI site of pCRISRPi as described elsewhere [51]. After transformation into *E*. *coli*, plasmid was purified from a transformant lacking fluorescence and silencing vectors were subsequently transformed into *S*. *aureus* JE2 as described elsewhere [51]. Gene knockdown was assessed using gene-targeted real-time PCR (**S2 Table**) of cDNA prepared from mid-log phase growth cells, normalizing gene-specific expression to that of *gyrA*. Gene expression levels in individual CRISPRi mutants were compared to relative gene expression obtained for a CRISRPi knockdown targeted to a biologically irrelevant sequence (GFP) [55] using the ΔΔCt method (**S3 Table**).

Quantitative macrophage pathogenesis phenotypes of CRISPRi mutants were assessed as above, but including 10 μg/mL chloramphenicol in all culture media to select for retention of plasmids, and comparing metrics to those of a GFP-silenced CRISPRi strain [56]. The GFP-silenced CRISPRi strain was found to be equivalent to those of the wild-type strain carrying a GFP-expressing plasmid built on the same vector backbone.

### Complementation studies

Genes for complementation studies were PCR amplified from JE2 genomic DNA (**S2 Table**) and cloned into vector pCN50wt. Where possible, the native promoters of cloned genes were used to drive gene expression by including the intergenic DNA falling between the gene of interest and the upstream gene. Three genes (*saeS*, B7H15_07805, and B7H15_05310) were assumed to be expressed within an operon on the basis of a gene in the same orientation terminating <30 bp upstream, and were therefore expressed under control of the constitutive *Pcap* promoter using PCR to add that element [57]. Transposon mutants were transformed with matched complementation vectors and with pCN50wt expressing superfolder GFP (ordered as a synthetic gene cassette, **S2 Table**) [58], which served as a comparator for phenotypic studies. Measurements of macrophage invasion and macrophage survival index were directly compared between complemented and GFP vector-transformed transposon mutant strains assayed in parallel, at least in quadruplicate.

### Macrophage viability assessments

The number of viable and dead macrophages was assessed 48 hours after *S*. *aureus* infection using tryptan blue exclusion assays as described elsewhere [59], with measurements taken at least in triplicate.

### Growth rate analyses

Growth rates were monitored by absorbance at OD_600_ during incubation at 37°C in sup-LB broth, using a BioTek Synergy H1 Plate reader (Agilent), with measurements taken in 10 minute intervals and experiments performed at least in triplicate.

## Results

### Intracellular pathogenesis of macrophages is elevated in *S*. *aureus* isolates from people with CF

We ascertained whether *S*. *aureus* from chronic airway infections in people with CF were subject to selection for enhanced intracellular macrophage pathogenesis *in vivo*. We utilized a previously described collection of clonally related *S*. *aureus* isolate pairs collected longitudinally over time from pediatric participants with CF [4], selecting a subset of 57 distinct clinical first- and last-collected isolate pairs that represented the genomic diversity present in the collection (**S1 Table**). To provide phylogenetically matched control strains for comparison, we compared each CF clinical isolate against a population of *S*. *aureus* control strains that were not derived from active disease and which originated from individuals without CF in order to identify those with the most closely matched genomes (n = 33).

CF isolates and lineage matched controls were assayed for intracellular pathogenesis phenotypes in differentiated THP-1 cells, which have previously been used as a model for *S*. *aureus* macrophage interactions [28,56,60]. Macrophage intracellular pathogenesis can be regarded either with respect to the proportion of bacteria that are able to enter and evade initial killing within host cells after phagocytosis (which in this study we term “invasion”), or to the rate of ongoing bacterial survival within host cells over longer periods of time (which here we term “survival”). We therefore assessed these properties separately: invasion was measured as the fraction of viable intracellular bacteria recovered from the initial inoculum 3 hours after sterilization of the extracellular compartment, whereas survival was assessed 48 hours after initial THP-1 host cell infection as the fraction of viable intracellular bacteria recovered at that time relative to the number ascertained at 3 hours.

**Fig 1:**
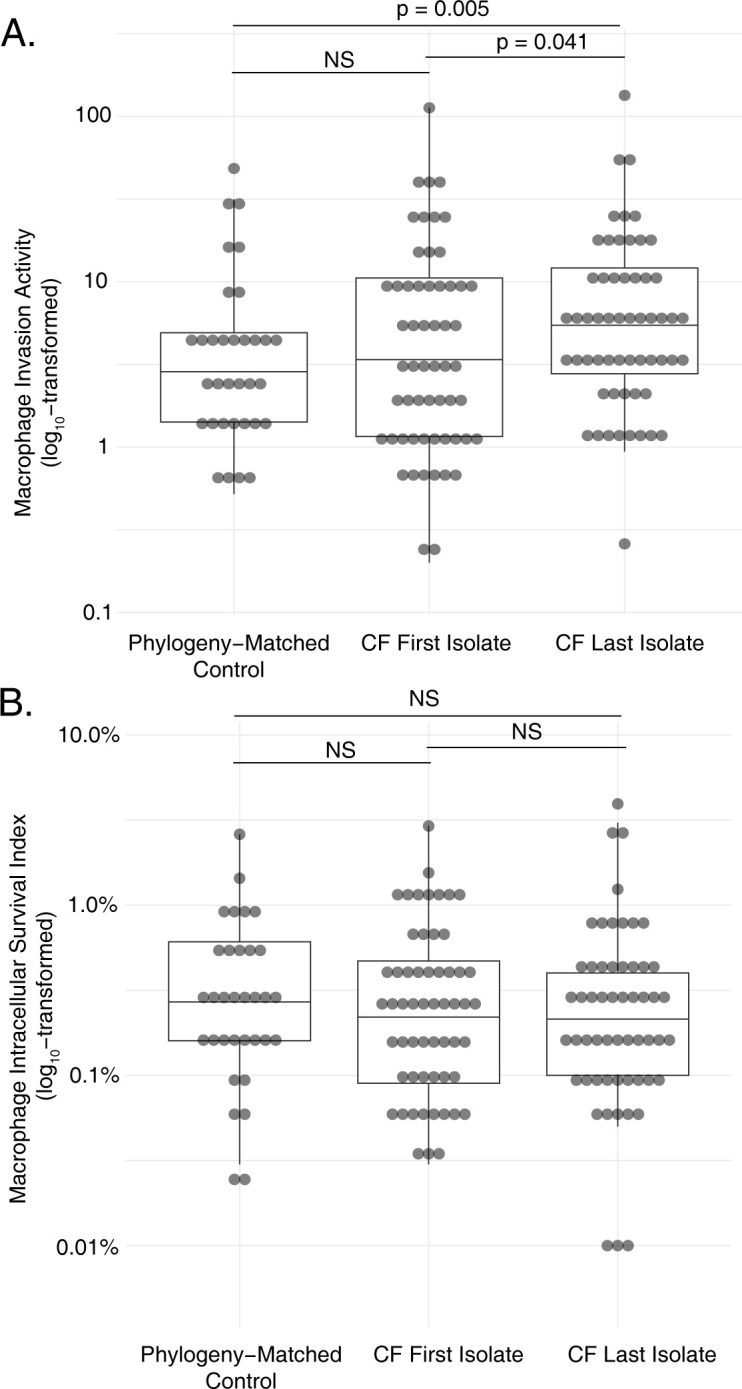
Macrophage pathogenesis phenotypes of *S*. *aureus* lineages from individuals with CF over time and phylogenetically matched control strains. Phenotypes of *S*. *aureus* strains isolated from respiratory cultures of individuals with CF at the time of initial isolation during serial surveillance are compared to the last-collected strains from same patient, and to phylogeny-matched strains from unaffected controls. Measures of macrophage invasion, relative to in-batch testing of the JE2 control strain (A), and macrophage intracellular survival (B) are separately reported. Statistical significance was assessed using paired two-tailed T tests.

We found that early *S*. *aureus* isolates obtained from individuals with CF by serial surveillance (i.e., those from the time of initial detection) exhibited capacities for macrophage invasion similar to those measured in phylogeny-matched control isolates from the general population. However, the final isolates collected from those same patient lineages after persistent respiratory infection (median 22.9 months, interquartile range 5.1 months), had significantly higher metrics of invasion than the early isolates (**Fig 1A**). 32 of the isolate pairs showed significantly (p<0.05, two-tailed T test) increased invasion phenotypes over time, while 14 demonstrated decreases, and 11 showed no significant change (**S1 Table and S2A Fig**). The aggregate increase in intracellular invasion phenotypes result was significant both when comparing groups of later isolates against the first clonally-related isolate collected from the same patient (p = 0.041, paired two-tailed T test) and against phylogeny matched controls from unaffected individuals (p = 0.005, paired two-tailed T test). In contrast, 20 of the isolate pairs showed significantly (p<0.05, two-tailed T test) increased levels of macrophage intracellular survival over time, while 20 evidenced decreases and 11 had no significant change in that phenotype. (**S1 Table and S2B Fig**). Differences between groups of first, last, and control isolates did not differ significantly among comparator populations in macrophage survival when considered in aggregate (**Fig 1B**).

These findings indicate that, at the time of initial detection, *S*. *aureus* strains infecting individuals with CF begin with capacities for macrophage invasion similar to strains from general population that are not involved in chronic disease, but that selective pressure for that phenotype occurs in the CF airway *in vivo*. Persistent survival within macrophages does not appear to be as strongly selected in CF respiratory infections, although it may be important in a subset of individual strains or clinical cases.

### Genomic analysis identifies candidate genes associated with intracellular macrophage pathogenesis *in vivo*

In order to identify genetic variation associated with enhanced macrophage pathogenesis phenotypes *in vivo*, we performed bacterial GWAS [48]. For both invasion and survival phenotypes, three complementary models of genetic association were applied (**S1 Fig**).

First we performed unitig-based testing, a reference-free method which identifies unique, variable-length sequences that are associated with a phenotype and incorporates statistical adjustment for bacterial population structure [61]. This analysis (**S1A Fig)** identified 179 sequences in 110 genes significantly associated with macrophage invasion **(S4 Table).** However, the unitigs most highly associated with macrophage invasion comprised an apparent haplotype, indicating insufficient sample diversity to resolve a specific candidate risk allele by this method and requiring prioritization of genes comprising multiple significantly associated unitigs for functional validation. No unitig sequences were significantly associated with survival.

Second, we conducted burden testing [62], which considers the collective impact of independent, non-synonymous variants occurring within a single annotated gene. This analysis (**S1B Fig, S5 Table and S6 Table)** identified five genes significantly associated with macrophage invasion (*purR*, *folB*, *sirA*, B7H15_03165, B7H15_06690) and three with macrophage survival (*recG*, B7H15_09405, B7H15_06245).

Last, we examined non-synonymous *de novo* mutations occurring between temporally first- and last-collected CF clinical isolates. This method of comparison provides the most robust control for bias related to residual population structure and reference annotation. This strategy (**S1C Fig)** identified 16 (**S7 Table**) and four genes (*clfA*, *fnbA*, *sdrE*, and a hypothetical protein, **S8 Table**) whose spontaneous mutation *in vivo* was associated with changes in macrophage invasion and survival, respectively. To assess the functional impact of such mutations, we examined the proportion of stop-gain mutations and indels (which are more likely to confer loss-of-function effects) to missense changes (which could confer carry either loss-of-function or gain-of-function effects) to assess for statistical imbalance between these classes for each gene. We found that three of the 16 invasion-associated genes (19%) and three of four of the survival-associated genes (75%) exhibited a statistically significant (p<0.05, Fisher Exact Test) excess of loss-of-function associated mutation types, although the missense mutations occurring in other genes may also exert similar effects. No genes demonstrated a proportional excess of missense mutation or patterns of mutation at common residues that would be expected from gain-of-function effects.

Together, these analyses implicate variation in 127 unique candidate genes as being correlated with macrophage pathogenesis *in vivo*, and suggest that mutations occurring *in vivo* frequently confer loss-of-function effects.

### Serial passaging selects mutants with elevated intracellular pathogenesis in macrophages *in vitro*

**Fig 2:**
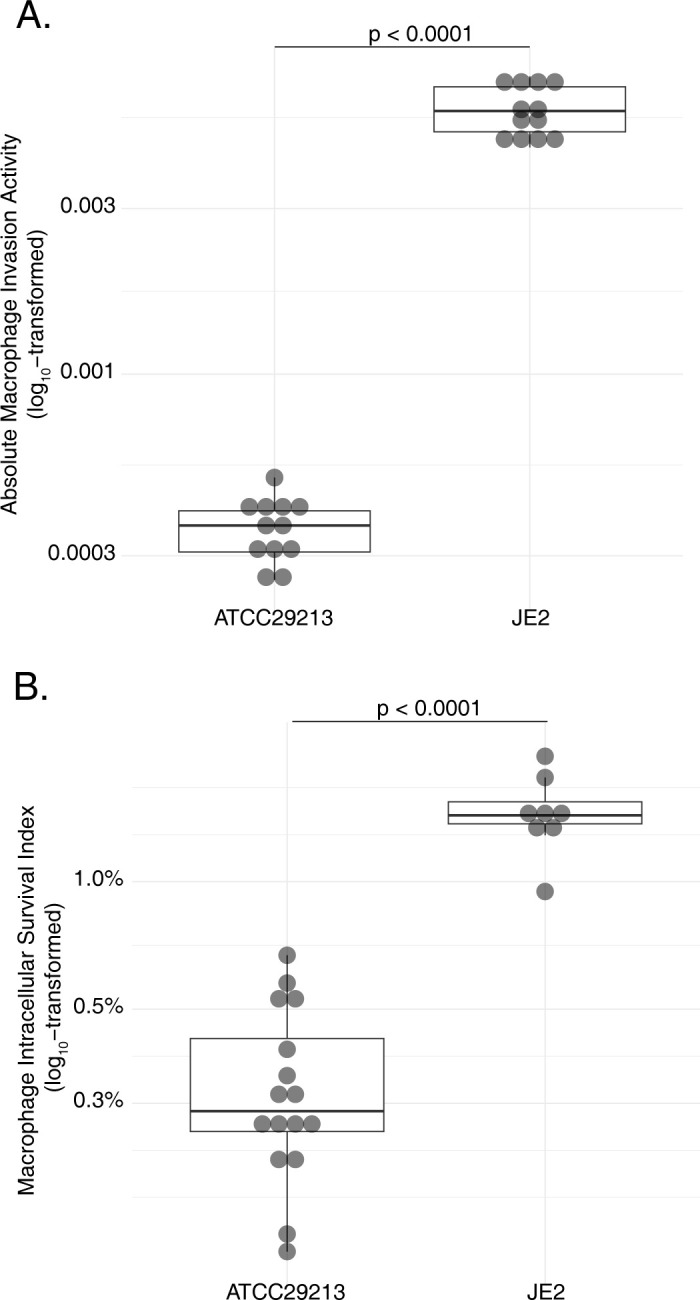
Macrophage pathogenesis phenotypes of *S*. *aureus* laboratory strains ATCC29213 and JE2. Phenotypes of strains were assessed for macrophage invasion (A) and macrophage intracellular survival (B). Statistical significance of measurements was assessed using unpaired two-tailed T tests. Macrophage invasion in this analysis was performed in a single experiment and determined as an absolute value, due to a lack of an external comparator strain for normalization.

To select for mutants having greater capacity to invade and survive within host macrophages under well-controlled laboratory conditions, and in well-defined bacterial genetic backgrounds, we separately conducted serial passaging experiments of *S*. *aureus* strains in the presence of THP-1 cell line-derived macrophages. We characterized the initial capacity for macrophage pathogenesis of two well-defined and phylogenomically distinct *S*. *aureus* reference strains, JE2 (Sequence Type 8, Clonal Complex 8) and ATCC29213 (Sequence Type 5, Clonal Complex 5). Viable bacteria from both strains were recovered three hours after macrophage invasion; however, a significantly greater proportion of JE2 were internalized at three hours relative to ATCC29213 (**Fig 2A**, p<0.0001, unpaired two-tailed T test). After normalizing for these differences in initial invasion, the natural capacity of these strains for ongoing intracellular survival at 48 hours also differed, with a significantly greater fraction of initially internalized JE2 surviving relative to ATCC29213 (**Fig 2B**, p<0.0001, unpaired two-tailed T test). To assess the fate of infected macrophages, we quantified their absolute number and the proportion of dead cells 48 hours after *S*. *aureus* was applied. Compared to an uninfected macrophage control, neither significant reduction in the number of macrophages (p>0.12, two-tailed T test) nor significant increases in the proportion of dead macrophages (p>0.45, two-tailed T test) were observed after infection with either *S*. *aureus* strain, indicating that *S*. *aureus* did not kill infected macrophages by lytic or non-lytic mechanisms.

From these differing natural baselines, six independent replicates from both strains were then passaged to select for intracellular macrophage pathogenesis phenotypes using lysostaphin protection assays [42,56], which selectively kill bacteria that have not been internalized by eukaryotic cells. After 12 passages, isolates were obtained from each expanded population and subjected to further characterization (**S9 Table**).

**Fig 3:**
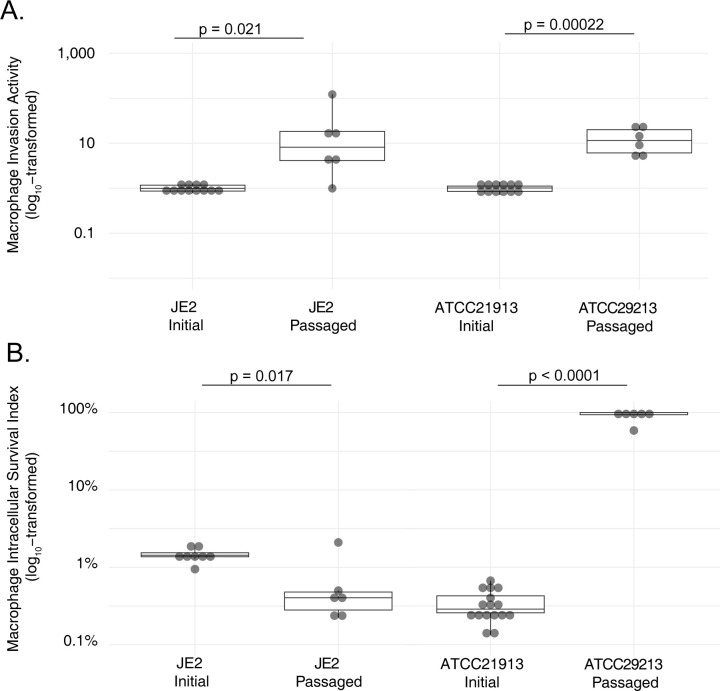
Macrophage pathogenesis phenotypes of passaged *S*. *aureus* laboratory strains. Derivatives of JE2 and ATCC29213 after passaging with selective pressure for macrophage pathogenesis assessed for macrophage invasion, relative to in-batch testing of the matched parental strain (A), and macrophage intracellular survival (B). Statistical significance was assessed using unpaired two-tailed T tests.

While strain JE2 showed greater baseline capacity for macrophage invasion than ATCC29213, that phenotype increased with passaging of both strains, derivatives of which ultimately achieved similar levels of activity (**Fig 3A**). In contrast, we observed significant strain-level differences in intracellular survival after selection (**Fig 3B**): ATCC29213, but not JE2, showed markedly increased survival in macrophages after passaging. For passaged ATCC29213 isolates, the fraction of bacteria recovered from macrophages after 48 hours exceeded that at the time of invasion, indicating some degree of bacterial replication following macrophage infection. The survival capacity of two JE2 derivatives decreased slightly with passaging, possibly indicating greater selective pressure for invasion phenotypes and mutual exclusivity of invasion and survival traits in that strain. Growth rate analysis of passaged mutants was conducted (**S10 Table)**. Half of the replicates (two ATCC29213 derivatives and four JE2 mutants) had growth rates not significantly different from their parental strains. Four replicates (three ATCC29213 and one JE2) had doubling times significantly greater than their parents, whereas only one replicate from each lineage replicated significantly faster.

Whole-genome sequencing detected a total of 15 genes that were mutated in passaged isolates (**S11 Table**). Most genes (n = 10) were mutated in only a single isolate, but five (*femA*, putative Abi-domain protease B7H15_05310, putative autolysin B7H15_09680, *vraS*, and *ccpA*) were each independently mutated in two to five evolved ATCC29213 isolates. Six of the mutated genes were affected by at least one unequivocally inactivating mutation (i.e., stop-gain or frameshift), consistent with loss-of-function effects, while the remainder were affected by missense mutations whose effects were less interpretable. No genes were mutated in common between ATCC29213 and JE2 over the course of passaging. Two JE2 replicates did not have detectable coding sequence or promoter mutations, suggesting epigenetic changes or mutations in remote regulatory elements.

### Comparison of candidate genes identified by *in vivo* and *in vitro* approaches

Among 127 candidate genes identified by GWAS and 15 identified by passaging, only a single gene (*clpX*) was implicated by both *in vivo* and *in vitro* approaches. Given that 5,854 unique genes were considered in the GWAS analysis, this degree of overlap did not exceed that expected by random chance (p = 0.228; significance threshold for number of overlapping genes for by SuperExactTest [63], method 2). We conclude that distinct gene subsets were identified by *in vivo* and *in vitro* analyses.

### Validation of candidate genes affecting macrophage intracellular pathogenesis

We next functionally evaluated candidate genes identified by *in vitro* and *in vivo* strategies for their ability to influence intracellular pathogenesis phenotypes in the THP-1 macrophage model. All genes identified by *in vitro* studies were targeted; however, given the large number of candidates implicated by analysis of *in vivo* isolates, we prioritized genes identified by GWAS for validation as those with the highest levels of association in a single analysis and those that were identified by more than one GWAS model. In total, the most significant 35 of 121 (28.9%) candidate invasion genes and all seven (100%) candidate survival genes met prioritization criteria for functional testing.

When available, we first tested the phenotypic impact of candidate genes using defined, isogenic transposon knockout mutants of strain JE2 [30]. Transposon mutants existed for 30 of 41 candidate genes prioritized from *in vivo* selection and 10 of the 15 of candidate *in vitro* genes. To further control for assay variability, we included transposon mutants for three genes that GWAS identified as having no association with intracellular pathogenesis, and which displayed similar intracellular pathogenesis phenotypes to the wild-type parental strain. We subsequently considered candidate genes to have a measurable impact on macrophage pathogenesis phenotypes if their corresponding transposon mutant effected a change that was statistically different from replicate measurements of the parental JE2 strain (defined as having a median measurement outside the 95% CI of controls).

**Fig 4:**
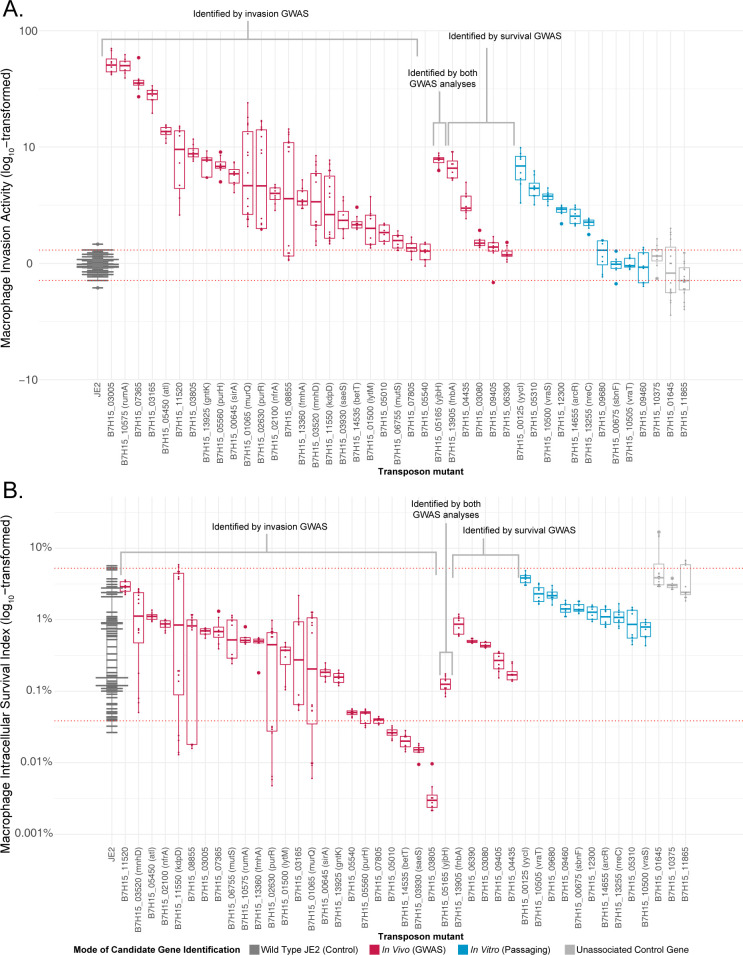
Transposon disruption of candidate macrophage pathogenesis genes. Fold-difference in measurements of macrophage invasion, relative to in-batch testing of the parental JE2 strain (A), and macrophage intracellular survival (B) for specified transposon mutants. Candidate genes are grouped according to the strategy by which they were identified, and sorted within groups by median value. Values from in-run JE2 strain controls are shown, and JE2 transposon mutants in genes not implicated in macrophage pathogenesis were used as an additional control group. Boxes indicate median, 25^th^, and 75^th^ percentiles with whiskers extending to the most distant value < 1.5 times the interquartile range. Red horizontal lines indicate the 95% confidence interval of all JE2 control measurements, with significant mutants defined as having a median measurement outside this range. Values higher than the 95% confidence intervals indicate increased macrophage pathogenesis phenotypes, and those below the interval correspond to decreased macrophage pathogenesis phenotypes. Genome-wide association study (GWAS).

Transposon disruption of 28 (93%) of *in vivo* candidates, six (60%) of *in vitro* candidates, and none of the uninvolved control genes significantly increased *S*. *aureus* invasion of THP-1 macrophages, while the remainder did not measurably alter that phenotype (**Fig 4A**). In contrast, *S*. *aureus* survival within macrophages was significantly decreased by disruption of only four (13%) of the *in vivo* candidates, none of the *in vitro* candidates, and none of the uninvolved control genes, with no enhancement of that phenotype observed (**Fig 4B**). Cumulatively, transposon disruptions of all but five candidate genes impacted macrophage pathogenesis. All such mutations improved macrophage invasion; however, four also negatively affected *S*. *aureus* survival within macrophages.

Given the large number of targets identified, we performed genetic complementation studies on a subset of genes showing the largest effect sizes for the invasion phenotype, and the complete, but more limited number of genes exhibiting significant survival effects (**S12 Table**). We transformed transposon mutant strains with appropriate complementation vectors and compared relevant phenotypes against matched controls transformed with a GFP-expressing plasmid. Complementation reverted the expected macrophage invasion phenotype in 12 of the 16 transposon mutants tested and the macrophage survival phenotype of three of the four mutants tested, providing further evidence for their biological impact. Failure of some mutants to yield the expected phenotype after complementation may reflect mismatches in the dosage or regulation of plasmid-borne genes, and thus does not necessarily indicate that the tested gene lacks contributions to macrophage pathogenesis.

To expand our scope of testing to factors not available as transposon knockouts, including potentially essential genes, we developed a constitutively expressed CRISPRi [31] gene silencing vector for use in *S*. *aureus*. Using high specificity guide RNA designs [54], nine additional isogenic knockdowns were successfully generated in JE2 (**S3 Table**) and their effects on intracellular pathogenesis assessed relative to CRISPRi knockdown of an irrelevant control target.

**Fig 5:**
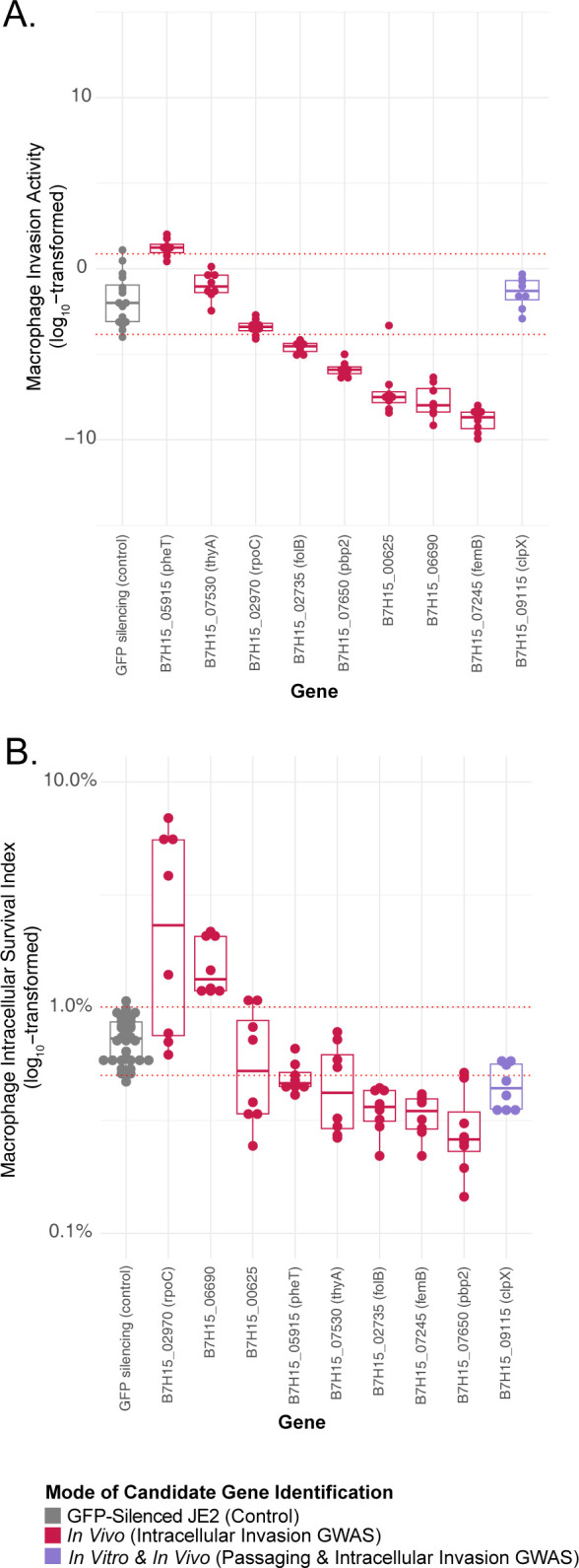
CRISPRi knockdown of candidate macrophage pathogenesis genes. Fold-difference in measurements of macrophage invasion, normalized to in-batch testing of the parental JE2 strain carrying a GFP-targeted CRISPRi construct(A), and macrophage intracellular survival (B) for specified CRISPRi gene knockdowns. Candidate genes are grouped according to the strategy by which they were identified, and sorted within groups by median value. JE2 carrying a GFP-expressing plasmid built on the same vector backbone as silencing constructs was used as an additional control group. Boxes indicate median, 25^th^, and 75^th^ percentiles with whiskers extending to the most distant value < 1.5 times the interquartile range. Red horizontal lines indicate the 95% confidence interval of all control measurements from JE2 with GFP-targeted CRISPRi, with significant mutants defined as having a median measurement outside this range. Values higher than the 95% confidence intervals indicate increased macrophage pathogenesis phenotypes, and those below the interval correspond to decreased macrophage pathogenesis phenotypes. Genome-wide association study (GWAS).

As anticipated, knockdowns did not quantitatively influence phenotypes as prominently as gene knockouts. Nevertheless, all tested candidate genes significantly impacted one or both macrophage pathogenesis phenotypes. One *in vivo* candidate gene significantly increased invasion and five others significantly decreased it (**Fig 5A**). Similarly, two of the *in vivo* candidates positively impacted macrophage survival, while five reduced it (**Fig 5B**). Knockdown of the single gene identified in common between *in vivo* analyses and passaging, *clpX*, also notably impaired survival.

Between the two validation approaches, 37 of the 39 tested candidate genes from *in vivo* analysis and seven of 11 genes from *in vitro* passaging impacted one or both macrophage pathogenesis phenotypes, with a single gene shared in common between those two sets.

### Features of *S*. *aureus* isogenic mutants with altered survival phenotypes

Counts of intracellular bacteria could be impacted by changes in bacterial survival, host-cell killing, or bacterial replicative capacity. We therefore assessed these features in isogenic mutants exhibiting differences in the macrophage survival phenotype. Relative to wild-type JE2, no mutant strain evidenced significant differences in the total number of macrophages (p>0.29, two-tailed T test) or the proportion of dead host cells (p>0.20, two-tailed T test) observed 48 hours after infection, indicating no measurable alterations to host cell death by lytic or non-lytic mechanisms. With only a single exception (*saeS*), isogenic mutants did not have significantly slower growth rates than matched comparator strains (**S13 Table**). We conclude that reduced intracellular survival phenotypes observed in isogenic mutants largely reflect differences in bacterial viability within macrophages, rather than reduced bacterial replication or impaired killing of the host cell population.

## Discussion

*S*. *aureus* must overcome different stressful conditions to establish and subsequently maintain an infection. During acute phases, bacteria must withstand innate immunity and destroy superficial host cells, whereas during chronic phases, persistent survival depends on resistance to lysosomal degradation and adaptation to low-nutrient conditions in intracellular environments [9]. Although some elements of phenotype switching [64] can be coordinated through changes in gene expression [27], prior work has shown that *S*. *aureus* undergoes genomic evolution (mutational adaptation) during infection to stably modify its initial phenotypes and better suit survival in the human host [4,65,66]. Here, we used parallel *in vivo* and *in vitro* experimental designs to identify genes contributing to two *S*. *aureus* phenotypes important to persistence: invasion, which is the ability to able to enter and survive within host cells after phagocytosis, and survival, the ability to persist intercellularly within macrophages over time.

Using chronic respiratory infection in people with CF as an exemplar of *S*. *aureus* persistence *in vivo*, we analyzed clonally related *S*. *aureus* isolate pairs taken from individuals early and late in the course of infection [4]. We found that the phenotypes of *S*. *aureus* isolated from patients with CF at the time of initial detection were equivalent to controls sharing a similar genetic background, consistent with the understanding that diverse *S*. *aureus* strains may be acquired from the community by individuals with CF without a requirement for specific virulence traits [4]. However, we observed that strains’ capacity for macrophage invasion increased significantly over time (**Fig 1A**), consistent with selective pressure for that feature being important during chronic *S*. *aureus* disease in individuals with CF [20,21]. In contrast, intracellular macrophage survival did not consistently increase during chronic infection, suggesting that trait may not be as universally selected. This could indicate that the inherent ability of *S*. *aureus* to survive within macrophages [1,67] is already adequate to sustain chronic persistence in CF [16,21,56], and that further improvements to that phenotype might incur disadvantageous fitness costs. Subsequent genome-wide association studies identified mutations in a total of 127 unique genes tracking with these phenotypic changes *in vivo* (**Fig 2**).

In parallel, we employed selection of laboratory *S*. *aureus* strains to model evolutionary pressures for macrophage pathogenesis *in vitro* (**Fig 4**) and used whole-genome analysis to identify associated mutations. Although that approach did not allow us to disambiguate mutations driving macrophage invasion from those influencing survival, it provided for well-controlled and well-defined experimental conditions. Whole-genome sequencing revealed 15 unique genes mutated in passaged isolates (**S11 Table**). Interestingly, we observed no overlap in the genes mutated during passaging of the two strains examined in this work, potentially indicating strain-specific mutation profiles or gene effects, consistent with our prior studies [56].

Using a combination of isogenic transposon mutant knockouts and CRISPRi knockdowns, we subsequently assessed the contributions of prioritized candidate genes identified by *in vitro* and *in vivo* analytic strategies (**Figs 4 and 5**). Forty-four of the 49 tested genes (90%) significantly affected one or both macrophage intracellular pathogenesis phenotypes. The majority of validated genes (n = 35, 80%) enhanced macrophage invasion when disrupted (**Table 1**), consistent with our finding that mutation of these genes is associated with increased macrophage pathogenesis *in vivo* (**Fig 1**) largely from the accumulation of loss-of-function variants. Only seven of the tested genes (14%) had an entirely negative phenotypic impact on *S*. *aureus* macrophage pathogenesis, although it is possible those factors may confer advantages *in vivo* that are not measured by our *in vitro* assay, or could prove beneficial when occurring in concert with secondary mutations or when affected by gain of function mutations not modeled in our validation.

**Table 1:** Validated genes in *S*. *aureus* impacting macrophage pathogenesis.

Category	Gene name or JE2 Locus ID	Gene Function	Evidence for macrophage pathogenesis	Identification strategy	Validation Strategy	Invasion Effect	Survival effect
metabolism	*arcR*	arginine deiminase operon repressor	[68]	*in vitro*	Knockout	Increase	NS†
	*purR* *	purine biosynthesis operon repressor	[69]	*in vivo* invasion	Knockout	Increase	NS
	*purH*	Bifunctional purine biosynthesis protein	[70]	*in vivo* invasion	Knockout	Increase	NS
	B7H15_07365	hydrolase	This study	*in vivo* invasion	Knockout	Increase	NS
	*kbl*	glycine C-acetyltransferase	This study	*in vivo* invasion	Knockout	Increase	NS
	*thiD*	hydroxymethylpyrimidine/phosphomethylpyrimidine kinase	This study	*in vivo* invasion	Knockout	Increase	NS
	*thyA*	thymidylate synthase	[71]	*in vivo* invasion	CRISPRi	NS	Decrease
	*cdr*	CoA-disulfide reductase	This study	*in vivo* invasion	Knockout	Increase	Decrease
	*pheT*	Phenylalanine—tRNA ligase beta subunit	This study	*in vivo* invasion	CRISPRi	Increase	Decrease
	*folB*	Dihydroneopterin aldolase	This study	*in vivo* invasion	CRISPRi	Decrease	Decrease
	*ansA*	L-asparaginase, type II	This study	*in vivo* invasion	Knockout	Increase	NS
	*ptaA*	PTS glucose transporter subunit IIBC	This study	*in vivo* survival	Knockout	Increase	NS
	*gntK*	Gluconokinase	This study	*in vivo* invasion	Knockout	Increase	NS
	*mnhD* *	Na+/H+ antiporter subunit D	This study	*in vivo* invasion	Knockout	Increase	NS
biofilm	*nreC*	Oxygen regulatory protein	[72]	*in vitro*	Knockout	Increase	NS
	*yycI* *	regulatory protein	[73]	*in vitro*	Knockout	Increase	NS
adherence and autolysis	*atl* *	bifunctional autolysin	[74]	*in vivo* invasion	Knockout	Increase	NS
	*fnbA* *	fibronectin-binding protein A	[75,76]	*in vivo* survival	Knockout	Increase	NS
	*clfA*	clumping factor A	[77]	*in vivo* survival	Knockout	Increase	NS
	*yycI* *	regulatory protein	[78]	*in vitro*	Knockout	Increase	NS
invasion	*atl* *	bifunctional autolysin	[79]	*in vivo* invasion	Knockout	Increase	NS
	*fnbA* *	fibronectin-binding protein A	[75,76]	*in vivo*	Knockout	Increase	NS
	B7H15_05310	putative Abi-domain protein	This study	*in vitro*	Knockout	Increase	NS
iron metabolism	*sirA*	iron ABC transporter substrate-binding protein	[80]	*in vivo* invasion	Knockout	Increase	NS
virulence gene regulation	*vraS*	two-component sensor histidine kinase	[81]	*in vitro*	Knockout	Increase	NS
	*kdpD*	sensor histidine kinase	[82]	*in vivo* invasion	Knockout	Increase	NS
	*saeS*	two-component sensor histidine kinase	[83–85]	*in vivo* invasion	Knockout	Increase	Decrease
	*purR* *	purine biosynthesis operon repressor	[69,86]	*in vivo* invasion	Knockout	Increase	NS
	*yjbH* *	transcriptional regulator	[87]	*in vivo* invasion *and survival*	Knockout	Increase	NS
	B7H15_06690	transcriptional regulator	This study	*in vivo* invasion	CRISPRi	Decrease	Increase
immune cell survival	*clpX*	protease	[89]	*in vitro*	CRISPRi	NS	Decrease
immune evasion	*sdrE*	MSCRAMM family adhesin	[88]	*in vivo* survival	Knockout	Increase	NS
	*lytM*	glycyl-glycine endopeptidase	This study	*in vivo* invasion	Knockout	Increase	NS
stress response	*yjbH* *	transcriptional regulator	[87]	*in vivo* invasion *and survival*	Knockout	Increase	NS
	*nfrA*	NADPH-dependent oxidoreductase	This study	*in vivo* invasion	Knockout	Increase	NS
	B7H15_12300	ClpA-like protein	This study	*in vitro*	Knockout	Increase	NS
	*mnhD* *	Na+/H+ antiporter subunit D	This study	*in vivo* invasion	Knockout	Increase	NS
	*betT*	choline transporter	This study	*in vivo* invasion	Knockout	Increase	Decrease
peptidoglycan metabolism	*fmhA*	methicillin resistance protein	This study	*in vivo* invasion	Knockout	Increase	NS
	*femA*	aminoacyltransferas	This study	*in vitro*	CRISPRi	Decrease	NS
	*femB*	aminoacyltransferase	This study	*in vivo* invasion	CRISPRi	Decrease	Decrease
	*pbp2*	penicillin-binding protein 2	This study	*in vivo* invasion	CRISPRi	Decrease	Decrease
mutation repair	*mutS*	DNA mismatch repair protein	This study	*in vivo* invasion	Knockout	Increase	NS
unknown	*murQ*	N-acetylmuramic acid 6-phosphate etherase	[90]	*in vivo* invasion	Knockout	Increase	NS
	*rumA*	23S rRNA (uracil(1939)-C(5))-methyltransferase	This study	*in vivo* invasion	Knockout	Increase	NS
	*rpoC*	DNA-directed RNA polymerase subunit beta	This study	*in vivo* invasion	CRISPRi	NS	Increase
	B7H15_00625	Hypothetical protein	This study	*in vivo* invasion	CRISPRi	Decrease	NS
	B7H15_08855	RecD/TraA family helicase	This study	*in vivo* invasion	Knockout	Increase	NS
	B7H15_03805	Hypothetical protein	This study	*in vivo* invasion	Knockout	Increase	Decrease
	B7H15_11520	Hypothetical protein	This study	*in vivo* invasion	Knockout	Increase	NS

* = gene with multiple functional categories

† NS = Not significant

Macrophage viability was not affected by the presence of infecting wild-type or mutant *S*. *aureus* strains, indicating that changes in macrophage pathogenesis reflect alterations in bacterial intracellular survival, rather than killing of the host-cell population. This is consistent with observations that *S*. *aureus* may not incur substantial macrophage death during infection [1,67].

Previously described virulence factors (n = 17) comprised 39% of all genes in our study having experimentally validated macrophage pathogenesis phenotypes, supporting the effectiveness of the overall experimental approach. These known factors contributed to diverse, well-described pathogenesis functions in CF and other diseases, comprising cellular metabolism (*arcR* [68], *purR* [69], *purH* [70], *thyA* [71]), biofilm formation (*nreC* [72], *yycI* [73]) and other adhesion mechanisms (*atl* [74], *fnbA* [75,76], *clfA* [77]), autolysis (*yycI* [78], *atl* [74]), invasins (*atl* [79], *fnbA* [75,76]), iron metabolism (*sirA* [80]), virulence gene regulation (*vraS* [81], *kdpD* [82], *saeS* [83–85], *purR* [69,86], *yjbH* [87]), and immune evasion (*sdrE* [88]). Other functions were more specialized to intracellular pathogenesis within immune cells, including protease *clpX* [89] and factors promoting protection against oxidative stress (*yjbH*(87)) which is a prominent feature of the macrophage intracellular environment. One virulence gene (*murQ*) has previously been reported to influence *S*. *aureus* hemolysis, by unknown mechanisms [90]. Notably, some virulence gene mutations that were found to positively influence macrophage invasion are known to attenuate *S*. *aureus* virulence in biological models (*purH*, *atl*, *fnbA*, *vraS*, *clpX*), illustrating that some mutations which are deleterious in acute infection states can be beneficial for chronic infection phenotypes.

A larger number of *S*. *aureus* macrophage pathogenesis genes (n = 27) are newly implicated by this effort, comprising 61% of genes passing experimental validation. The putative or documented roles of many mirror the functions of known virulence factors, including Abi-domain protein (B7H15_05310 [91]), immune evasion (*lytM* [92,93]), transcriptional regulation (B7H15_06690), cellular metabolism (*thiD*, *ptaA*, *pheT*, *folB*, *gntK*, *mnhD* [94], *kbl*, *cdr*, *ansA*, B7H15_07365), and response to oxidative (*nfrA* [95], *clpA* [96]-like gene B7H15_12300), pH (*mnhD* [97]), or osmotic (*betT* [98]) stress. However, other newly validated genes offer insight into previously unappreciated mechanisms affecting intracellular macrophage pathogenesis. The most substantial constellation of functionally related genes include *fmhA* [99], *femA* [100], *femB* [100], and *pbp2* [100], all of which have functions in peptidoglycan assembly. This finding potentially indicates an important role for cell wall metabolism in *S*. *aureus* persistence, either due to direct changes in host cell interaction [101–103], or specific structural changes that modulate fitness within macrophages [104]. The well-characterized mismatch repair gene *mutS* conferred increased invasion phenotypes when inactivated by transposon mutagenesis, potentially reflecting greater population diversity carrying phenotypically advantageous mutations [105] and not acting as a virulence factor directly. Although the remaining validated genes had hypothetical functions or non-obvious roles in pathogenesis, some, such as *rumA*, had pronounced phenotypic effects and merit dedicated investigations to understand their contributions to *S*. *aureus* macrophage pathogenesis.

In addition to illuminating previously overlooked factors relevant to intracellular macrophage pathogenesis and chronic infection, these studies highlight pronounced differences between the results of *in vivo* and *in vitro* investigatory strategies. Macrophage pathogenesis phenotypes quantitatively increased during both chronic human infection and serial passaging, and most genes implicated by *in vivo* and *in vitro* strategies were confirmed to impact macrophage pathogenesis in the same functional laboratory model. Yet, despite these phenotypic similarities, only a single candidate gene was identified in common by parallel *in vivo* and *in vitro* studies. Although some disparities could be attributed to strain-specific differences [56], the consistency of particular mutations occurring *in vivo* among independent strains from different patients argues against this phenomenon being widespread. We conclude that mutations identified even in highly controlled *in vitro* studies may fail to identify changes enhancing the same phenotypes that arise naturally in human disease. This schism, which we previously observed in the evolution of *P*. *aeruginosa* antibiotic resistance [106], may reflect differences in fitness constraints, selective pressures, and population sizes occurring *in vivo* and *in vitro*. Although both strategies identify genes measurably contributing to pathogenesis in our cultured macrophage model, this finding illustrates the value of analyzing clinical isolates to understand factors driving infectious disease *in vivo*.

Our work is subject to several limitations. Cell lines cultured *in vitro* are not physically or biochemically identical to analogous cells or tissues found *in vivo* [34], and macrophages from individuals with CF have some impaired functionality [21,107], making both our *in vitro* selection schema and macrophage pathogenesis assays necessarily contrived. However, our finding that metrics of THP-1 invasion employed here and by others [28,60] increased during chronic infection offers support that the model reasonably approximates *in vivo* conditions and is clinically relevant. Separately, our approach may not identify legitimate, but less frequently occurring mutational adaptations due to the sample sizes available. Effector genes whose contributions to intracellular pathogenesis are not quantitatively large may have similarly been overlooked by our strict functional validation criteria. It is also possible that critical adaptations in our *in vivo* study population occurred prior to collection of the earliest CF isolates, limiting identification of key genes that are subject to rapid selection: however, functional measures of intracellular pathogenesis in these first isolates were similar to phylogenetically matched control strains not associated with infection, indicating that the impact of early adaptation not captured in this study was small. Additionally, CF isolates were derived from sputum and oropharyngeal swabs, as bronchoalveolar lavage is not commonly performed in children, and thereby may not fully reflect the microbiology of the lower airways [4]. Lastly, we are unable to isolate specific selective pressures occurring *in vivo* or to correlate them with isolate phenotypes: *S*. *aureus* is able to invade and infect a wide variety of cell types [12–15,21], and it is possible that selective pressures for invasion of non-phagocytic host cells, or less likely, for phenotypes unrelated to host cell invasion, occur *in vivo* and the enhancement of macrophage invasion is merely coincidental.

Although this effort focused on a model of respiratory infection in CF, *S*. *aureus* is capable of causing infection in a variety of organ systems [7,14,19,108,109] and host-cell invasion is a hallmark of many *S*. *aureus* chronic disease states [12–15,21]. The effector genes identified here may therefore have generalized importance in other infective processes, and indeed, show activity in a macrophage cell line that is not affected by *CFTR* mutation. Future work will seek to more comprehensively catalog the intracellular pathogenesis mechanisms present across *S*. *aureus* genetic lineages and to understand similarities and differences in the gene content promoting pathogenesis of additional host cell types. This knowledge may enable development of novel therapeutic strategies to prevent the establishment of bacterial intracellular reservoirs or to disrupt persistent populations that sustain chronic infection and that are resistant to conventional forms of treatment.

## Supporting information

S1 Table S. aureus clinical strains and properties. (XLSX)

S2 Table Oligo sequences. (XLSX)

S3 Table CRISPRi gene knockdown results. (XLSX)

S4 Table Unitig genome-wide association study results for invasion. (XLSX)

S5 Table Burden testing genome-wide association study results for invasion. (XLSX)

S6 Table Burden testing genome-wide association study results for survival. (XLSX)

S7 Table First-last isolate pair analysis for invasion. (XLSX)

S8 Table First-last isolate pair analysis for survival. (XLSX)

S9 Table Macrophage pathogenesis phenotypes of passaged S. aureus replicates. (XLSX)

S10 Table Growth rates of passaged isolates. (XLSX)

S11 Table Gene mutations identified in passaged S. aureus replicates. (XLSX)

S12 Table Phenotypes of complemented transposon mutants. (XLSX)

S13 Table Growth rates of isogenic mutants with altered macrophage survival phenotypes. (XLSX)

S1 Fig Genome-wide association study results for macrophage pathogenesis in vivo. Quantile-quantile plots for *in vivo* mutations associated with macrophage invasion (blue) and survival (red) phenotypes by various implementations of genome-wide association studies. Association between phenotypes by analysis of (**A**) reference-free sequence differences (“unitigs”), (**B**) non-synonymous mutations with gene kernels (“burden testing”), and (**C**) *de novo* mutations arising within clonally related patient lineages over time (“first-last comparison”). Significant and non-significant results are shaded differentially.
(PDF)

S2 Fig Changes in macrophage pathogenesis phenotypes between individual first-last clinical isolate pairs. Phenotypes of *S*. *aureus* strains isolated from respiratory cultures of individuals with CF at the time of initial isolation during serial surveillance are compared to the last-collected strains from same patient. Colored lines in this connected dot plot indicate the direction and significance of changes in individual first-last isolate pairs (red = significant increase over time, blue = significant decrease over time, grey = no-significant change) for measures of macrophage invasion relative to in-batch testing of the JE2 control strain (A), and macrophage intracellular survival (B). Statistical significance was assessed using paired two-tailed T tests.
(PDF)

## References

[1] Pidwill GR, Gibson JF, Cole J, Renshaw SA, Foster SJ. The Role of Macrophages in Staphylococcus aureus Infection. Front Immunol. 2021 Jan 19;11:620339. doi: 10.3389/fimmu.2020.620339 PMC7850989 33542723

[2] Hirschhausen N, Block D, Bianconi I, Bragonzi A, Birtel J, Lee JC, et al. Extended Staphylococcus aureus persistence in cystic fibrosis is associated with bacterial adaptation. Int J Med Microbiol. 2013 Dec;303(8):685–92. doi: 10.1016/j.ijmm.2013.09.012 24183484

[3] Branger C, Gardye C, Lambert-Zechovsky N. Persistence of Staphylococcus aureus strains among cystic fibrosis patients over extended periods of time. J Med Microbiol. 1996 Oct;45(4):294–301. doi: 10.1099/00222615-45-4-294 8849704

[4] Long DR, Wolter DJ, Lee M, Precit M, McLean K, Holmes E, et al. Polyclonality, Shared Strains, and Convergent Evolution in Chronic Cystic Fibrosis Staphylococcus aureus Airway Infection. Am J Respir Crit Care Med. 2021 May 1;203(9):1127–37. doi: 10.1164/rccm.202003-0735OC 33296290 PMC8314914

[5] Al-Zubeidi D, Hogan PG, Boyle M, Burnham CAD, Fritz SA. Molecular epidemiology of methicillin-resistant Staphylococcus aureus isolated in serial cultures from the respiratory tract of children with cystic fibrosis. Pediatr Infect Dis J. 2014 Jun;33(6):549–53. doi: 10.1097/INF.0000000000000204 24220228 PMC4016999

[6] Andersen C, Kahl BC, Olesen HV, Jensen-Fangel S, Nørskov-Lauritsen N. Intravenous antibiotics given for 2 weeks do not eradicate persistent Staphylococcus aureus clones in cystic fibrosis patients. Clin Microbiol Infect. 2014 May;20(5):O285–291. doi: 10.1111/1469-0691.12406 24112282

[7] Conlon BP. Staphylococcus aureus chronic and relapsing infections: Evidence of a role for persister cells: An investigation of persister cells, their formation and their role in S. aureus disease. Bioessays. 2014 Oct;36(10):991–6. doi: 10.1002/bies.201400080 25100240

[8] Goerke C, Wolz C. Adaptation of Staphylococcus aureus to the cystic fibrosis lung. Int J Med Microbiol. 2010 Dec;300(8):520–5. doi: 10.1016/j.ijmm.2010.08.003 20843740

[9] Tuchscherr L, Bischoff M, Lattar SM, Noto Llana M, Pförtner H, Niemann S, et al. Sigma Factor SigB Is Crucial to Mediate Staphylococcus aureus Adaptation during Chronic Infections. PLoS Pathog. 2015 Apr;11(4):e1004870. doi: 10.1371/journal.ppat.1004870 25923704 PMC4414502

[10] Suligoy CM, Lattar SM, Noto Llana M, González CD, Alvarez LP, Robinson DA, et al. Mutation of Agr Is Associated with the Adaptation ofStaphylococcus aureusto the Host during Chronic Osteomyelitis. Front Cell Infect Microbiol. 2018;8:18. doi: 10.3389/fcimb.2018.00018 29456969 PMC5801681

[11] Howden BP, Davies JK, Johnson PDR, Stinear TP, Grayson ML. Reduced Vancomycin Susceptibility in Staphylococcus aureus, Including Vancomycin-Intermediate and Heterogeneous Vancomycin-Intermediate Strains: Resistance Mechanisms, Laboratory Detection, and Clinical Implications. Clinical Microbiology Reviews. 2010 Jan 1;23(1):99–139. 20065327 10.1128/CMR.00042-09 PMC2806658

[12] Fraunholz M, Sinha B. Intracellular Staphylococcus aureus: live-in and let die. Front Cell Infect Microbiol. 2012;2:43. doi: 10.3389/fcimb.2012.00043 22919634 PMC3417557

[13] Lowy FD. Is Staphylococcus aureus an intracellular pathogen? Trends Microbiol. 2000 Aug;8(8):341–3. doi: 10.1016/s0966-842x(00)01803-5 10920387

[14] Garzoni C, Kelley WL. Staphylococcus aureus: new evidence for intracellular persistence. Trends Microbiol. 2009 Feb;17(2):59–65. doi: 10.1016/j.tim.2008.11.005 19208480

[15] Moldovan A, Fraunholz MJ. In or out: Phagosomal escape of *Staphylococcus aureus*. Cellular Microbiology. 2019 Mar;21(3):e12997. 30576050 10.1111/cmi.12997

[16] Lacoma A, Cano V, Moranta D, Regueiro V, Domínguez-Villanueva D, Laabei M, et al. Investigating intracellular persistence of Staphylococcus aureus within a murine alveolar macrophage cell line. Virulence. 2017 Nov 17;8(8):1761–75. doi: 10.1080/21505594.2017.1361089 28762868 PMC5810471

[17] Thwaites GE, Gant V. Are bloodstream leukocytes Trojan Horses for the metastasis of Staphylococcus aureus? Nat Rev Microbiol. 2011 Mar;9(3):215–22. doi: 10.1038/nrmicro2508 21297670

[18] Kubica M, Guzik K, Koziel J, Zarebski M, Richter W, Gajkowska B, et al. A potential new pathway for Staphylococcus aureus dissemination: the silent survival of S. aureus phagocytosed by human monocyte-derived macrophages. PLoS ONE. 2008 Jan 9;3(1):e1409. doi: 10.1371/journal.pone.0001409 18183290 PMC2169301

[19] Kalinka J, Hachmeister M, Geraci J, Sordelli D, Hansen U, Niemann S, et al. Staphylococcus aureus isolates from chronic osteomyelitis are characterized by high host cell invasion and intracellular adaptation, but still induce inflammation. Int J Med Microbiol. 2014 Nov;304(8):1038–49. doi: 10.1016/j.ijmm.2014.07.013 25129555

[20] Tan X, Coureuil M, Ramond E, Euphrasie D, Dupuis M, Tros F, et al. Chronic Staphylococcus aureus Lung Infection Correlates With Proteogenomic and Metabolic Adaptations Leading to an Increased Intracellular Persistence. Clinical Infectious Diseases. 2019 Nov 13;69(11):1937–45. doi: 10.1093/cid/ciz106 30753350

[21] Li C, Wu Y, Riehle A, Ma J, Kamler M, Gulbins E, et al. Staphylococcus aureus Survives in Cystic Fibrosis Macrophages, Forming a Reservoir for Chronic Pneumonia. Infect Immun. 2017 May;85(5). doi: 10.1128/IAI.00883-16 28289144 PMC5400852

[22] Trouillet-Assant S, Lelièvre L, Martins-Simões P, Gonzaga L, Tasse J, Valour F, et al. Adaptive processes of *Staphylococcus aureus* isolates during the progression from acute to chronic bone and joint infections in patients: S. aureus adaptation during BJI. Cellular Microbiology. 2016 Oct;18(10):1405–14. 26918656 10.1111/cmi.12582

[23] Siwczak F, Cseresnyes Z, Hassan MIA, Aina KO, Carlstedt S, Sigmund A, et al. Human macrophage polarization determines bacterial persistence of Staphylococcus aureus in a liver-on-chip-based infection model. Biomaterials. 2022 Aug;287:121632. doi: 10.1016/j.biomaterials.2022.121632 35728409

[24] Hamza T, Dietz M, Pham D, Clovis N, Danley S, Li B. Intra-cellular Staphylococcus aureus alone causes infection in vivo. Eur Cell Mater. 2013 Jul 8;25:341–50; discussion 350. doi: 10.22203/ecm.v025a24 23832687 PMC3830899

[25] Plouin-Gaudon I, Clement S, Huggler E, Chaponnier C, François P, Lew D, et al. Intracellular residency is frequently associated with recurrent Staphylococcus aureus rhinosinusitis. Rhinology. 2006 Dec;44(4):249–54. 17216740

[26] Vozza EG, Mulcahy ME, McLoughlin RM. Making the Most of the Host; Targeting the Autophagy Pathway Facilitates Staphylococcus aureus Intracellular Survival in Neutrophils. Front Immunol. 2021 Jun 16;12:667387. doi: 10.3389/fimmu.2021.667387 34220813 PMC8242348

[27] Soe YM, Bedoui S, Stinear TP, Hachani A. Intracellular *Staphylococcus aureus* and host cell death pathways. Cellular Microbiology [Internet]. 2021 May [cited 2023 Aug 21];23(5). Available from: https://onlinelibrary.wiley.com/doi/10.1111/cmi.13317. 10.1111/cmi.13317 33550697

[28] Nguyen HA, Denis O, Vergison A, Theunis A, Tulkens PM, Struelens MJ, et al. Intracellular Activity of Antibiotics in a Model of Human THP-1 Macrophages Infected by a *Staphylococcus aureus* Small-Colony Variant Strain Isolated from a Cystic Fibrosis Patient: Pharmacodynamic Evaluation and Comparison with Isogenic Normal-Phenotype and Revertant Strains. Antimicrob Agents Chemother. 2009 Apr;53(4):1434–42. 19188393 10.1128/AAC.01145-08 PMC2663071

[29] Chanput W, Mes JJ, Wichers HJ. THP-1 cell line: an in vitro cell model for immune modulation approach. Int Immunopharmacol. 2014 Nov;23(1):37–45. doi: 10.1016/j.intimp.2014.08.002 25130606

[30] Fey PD, Endres JL, Yajjala VK, Widhelm TJ, Boissy RJ, Bose JL, et al. A genetic resource for rapid and comprehensive phenotype screening of nonessential Staphylococcus aureus genes. MBio. 2013 Feb 12;4(1):e00537–00512. doi: 10.1128/mBio.00537-12 23404398 PMC3573662

[31] Larson MH, Gilbert LA, Wang X, Lim WA, Weissman JS, Qi LS. CRISPR interference (CRISPRi) for sequence-specific control of gene expression. Nat Protoc. 2013 Nov;8(11):2180–96. doi: 10.1038/nprot.2013.132 24136345 PMC3922765

[32] Precit MR, Wolter DJ, Griffith A, Emerson J, Burns JL, Hoffman LR. Optimized In Vitro Antibiotic Susceptibility Testing Method for Small-Colony Variant Staphylococcus aureus. Antimicrob Agents Chemother. 2016 Jan 4;60(3):1725–35. doi: 10.1128/AAC.02330-15 26729501 PMC4775967

[33] Dantes R, Mu Y, Belflower R, Aragon D, Dumyati G, Harrison LH, et al. National burden of invasive methicillin-resistant Staphylococcus aureus infections, United States, 2011. JAMA Intern Med. 2013 Nov 25;173(21):1970–8. 10.1001/jamainternmed.2013.10423 PMC10887428 24043270

[34] Strobel M, Pförtner H, Tuchscherr L, Völker U, Schmidt F, Kramko N, et al. Post-invasion events after infection with Staphylococcus aureus are strongly dependent on both the host cell type and the infecting S. aureus strain. Clin Microbiol Infect. 2016 Sep;22(9):799–809. 27393124 10.1016/j.cmi.2016.06.020

[35] Jackman SD, Vandervalk BP, Mohamadi H, Chu J, Yeo S, Hammond SA, et al. ABySS 2.0: resource-efficient assembly of large genomes using a Bloom filter. Genome Res. 2017 May;27(5):768–77. doi: 10.1101/gr.214346.116 28232478 PMC5411771

[36] Page AJ, Cummins CA, Hunt M, Wong VK, Reuter S, Holden MTG, et al. Roary: rapid large-scale prokaryote pan genome analysis. Bioinformatics. 2015 Nov 15;31(22):3691–3. doi: 10.1093/bioinformatics/btv421 26198102 PMC4817141

[37] Page AJ, Taylor B, Delaney AJ, Soares J, Seemann T, Keane JA, et al. SNP-sites: rapid efficient extraction of SNPs from multi-FASTA alignments. Microb Genom. 2016 Apr;2(4):e000056. doi: 10.1099/mgen.0.000056 28348851 PMC5320690

[38] Stuart EA. Matching methods for causal inference: A review and a look forward. Stat Sci. 2010 Feb 1;25(1):1–21. doi: 10.1214/09-STS313 20871802 PMC2943670

[39] Tsuchiya S, Kobayashi Y, Goto Y, Okumura H, Nakae S, Konno T, et al. Induction of maturation in cultured human monocytic leukemia cells by a phorbol diester. Cancer Res. 1982 Apr;42(4):1530–6. 6949641

[40] Aldo PB, Craveiro V, Guller S, Mor G. Effect of culture conditions on the phenotype of THP-1 monocyte cell line. Am J Reprod Immunol. 2013 Jul;70(1):80–6. doi: 10.1111/aji.12129 23621670 PMC3703650

[41] Maeß MB, Wittig B, Cignarella A, Lorkowski S. Reduced PMA enhances the responsiveness of transfected THP-1 macrophages to polarizing stimuli. J Immunol Methods. 2014 Jan 15;402(1–2):76–81. doi: 10.1016/j.jim.2013.11.006 24269601

[42] Kim JH, Chaurasia AK, Batool N, Ko KS, Kim KK. Alternative Enzyme Protection Assay To Overcome the Drawbacks of the Gentamicin Protection Assay for Measuring Entry and Intracellular Survival of Staphylococci. Infect Immun. 2019 Mar;87(5):e00119–19. doi: 10.1128/IAI.00119-19 30782857 PMC6479035

[43] Werth BJ, Ashford NK, Penewit K, Waalkes A, Holmes EA, Bryan A, et al. Evolution of cefiderocol resistance in Stenotrophomonas maltophilia using in vitro serial passage techniques. JAC Antimicrob Resist. 2022 Mar;4(1):dlac011. doi: 10.1093/jacamr/dlac011 35156034 PMC8827560

[44] Robinson JT, Thorvaldsdóttir H, Winckler W, Guttman M, Lander ES, Getz G, et al. Integrative genomics viewer. Nat Biotechnol. 2011 Jan;29(1):24–6. doi: 10.1038/nbt.1754 21221095 PMC3346182

[45] Altschul SF, Gish W, Miller W, Myers EW, Lipman DJ. Basic local alignment search tool. J Mol Biol. 1990 Oct 5;215(3):403–10. doi: 10.1016/S0022-2836(05)80360-2 2231712

[46] Fuchs S, Mehlan H, Bernhardt J, Hennig A, Michalik S, Surmann K, et al. Aureo Wiki – The repository of the Staphylococcus aureus research and annotation community. International Journal of Medical Microbiology. 2018 Aug;308(6):558–68. 29198880 10.1016/j.ijmm.2017.11.011

[47] Jolley KA, Maiden MC. BIGSdb: Scalable analysis of bacterial genome variation at the population level. BMC Bioinformatics. 2010 Dec;11(1):595. 21143983 10.1186/1471-2105-11-595 PMC3004885

[48] Lees JA, Galardini M, Bentley SD, Weiser JN, Corander J. pyseer: a comprehensive tool for microbial pangenome-wide association studies. Bioinformatics. 2018 Dec 15;34(24):4310–2. doi: 10.1093/bioinformatics/bty539 30535304 PMC6289128

[49] Price MN, Dehal PS, Arkin AP. FastTree 2 –Approximately Maximum-Likelihood Trees for Large Alignments. Poon AFY, editor. PLoS ONE. 2010 Mar 10;5(3):e9490. doi: 10.1371/journal.pone.0009490 20224823 PMC2835736

[50] Long DR, Penewit K, Lo HY, Almazan J, Holmes EA, Bryan AB, et al. In Vitro Selection Identifies Staphylococcus aureus Genes Influencing Biofilm Formation. Infect Immun. 2023 Mar 15;91(3):e0053822. doi: 10.1128/iai.00538-22 36847490 PMC10016075

[51] Penewit K, Holmes EA, McLean K, Ren M, Waalkes A, Salipante SJ. Efficient and Scalable Precision Genome Editing inStaphylococcus aureusthrough Conditional Recombineering and CRISPR/Cas9-Mediated Counterselection. MBio. 2018 Feb 20;9(1). 10.1128/mBio.00067-18 PMC5821094 29463653

[52] Bikard D, Jiang W, Samai P, Hochschild A, Zhang F, Marraffini LA. Programmable repression and activation of bacterial gene expression using an engineered CRISPR-Cas system. Nucleic Acids Res. 2013 Aug;41(15):7429–37. doi: 10.1093/nar/gkt520 23761437 PMC3753641

[53] Lewis JD, Salipante SJ. Development of advanced control material for reverse transcription-mediated bacterial nucleic acid amplification tests. J Clin Microbiol. 2024 May 8;62(5):e0024324. doi: 10.1128/jcm.00243-24 38629844 PMC11237385

[54] Concordet JP, Haeussler M. CRISPOR: intuitive guide selection for CRISPR/Cas9 genome editing experiments and screens. Nucleic Acids Research. 2018 Jul 2;46(W1):W242–5. doi: 10.1093/nar/gky354 29762716 PMC6030908

[55] de Bakker V, Liu X, Bravo AM, Veening JW. CRISPRi-seq for genome-wide fitness quantification in bacteria. Nat Protoc. 2022 Feb;17(2):252–81. doi: 10.1038/s41596-021-00639-6 34997243

[56] Lo HY, Long DR, Holmes EA, Penewit K, Hodgson T, Lewis JD, et al. Transposon sequencing identifies genes impacting Staphylococcus aureus invasion in a human macrophage model. Infect Immun. 2023 Oct 17;91(10):e0022823. doi: 10.1128/iai.00228-23 37676013 PMC10580828

[57] Schwendener S, Perreten V. New shuttle vector-based expression system to generate polyhistidine-tagged fusion proteins in Staphylococcus aureus and Escherichia coli. Appl Environ Microbiol. 2015 May 1;81(9):3243–54. doi: 10.1128/AEM.03803-14 25747000 PMC4393442

[58] Pédelacq JD, Cabantous S, Tran T, Terwilliger TC, Waldo GS. Engineering and characterization of a superfolder green fluorescent protein. Nat Biotechnol. 2006 Jan;24(1):79–88. doi: 10.1038/nbt1172 16369541

[59] Strober W. Trypan Blue Exclusion Test of Cell Viability. Curr Protoc Immunol. 2015 Nov 2;111:A3.B.1-A3.B.3. 10.1002/0471142735.ima03bs111 PMC6716531 26529666

[60] Gabryszewski SJ, Wong Fok Lung T, Annavajhala MK, Tomlinson KL, Riquelme SA, Khan IN, et al. Metabolic Adaptation in Methicillin-Resistant Staphylococcus aureus Pneumonia. Am J Respir Cell Mol Biol. 2019 Aug;61(2):185–97. doi: 10.1165/rcmb.2018-0389OC 30742488 PMC6670030

[61] San JE, Baichoo S, Kanzi A, Moosa Y, Lessells R, Fonseca V, et al. Current Affairs of Microbial Genome-Wide Association Studies: Approaches, Bottlenecks and Analytical Pitfalls. Front Microbiol. 2020 Jan 30;10:3119. doi: 10.3389/fmicb.2019.03119 32082269 PMC7002396

[62] Lee S, Abecasis GR, Boehnke M, Lin X. Rare-variant association analysis: study designs and statistical tests. Am J Hum Genet. 2014 Jul 3;95(1):5–23. doi: 10.1016/j.ajhg.2014.06.009 24995866 PMC4085641

[63] Wang M, Zhao Y, Zhang B. Efficient Test and Visualization of Multi-Set Intersections. Sci Rep. 2015 Nov 25;5(1):16923. doi: 10.1038/srep16923 26603754 PMC4658477

[64] Edwards AM. Phenotype switching is a natural consequence of Staphylococcus aureus replication. J Bacteriol. 2012 Oct;194(19):5404–12. doi: 10.1128/JB.00948-12 22865841 PMC3457229

[65] Proctor RA, Balwit JM, Vesga O. Variant subpopulations of Staphylococcus aureus as cause of persistent and recurrent infections. Infect Agents Dis. 1994 Dec;3(6):302–12. 7889317

[66] Fitzgerald JR. Evolution of Staphylococcus aureus during human colonization and infection. Infect Genet Evol. 2014 Jan;21:542–7. doi: 10.1016/j.meegid.2013.04.020 23624187

[67] Koziel J, Maciag-Gudowska A, Mikolajczyk T, Bzowska M, Sturdevant DE, Whitney AR, et al. Phagocytosis of Staphylococcus aureus by macrophages exerts cytoprotective effects manifested by the upregulation of antiapoptotic factors. PLoS ONE. 2009;4(4):e5210. doi: 10.1371/journal.pone.0005210 19381294 PMC2668171

[68] Makhlin J, Kofman T, Borovok I, Kohler C, Engelmann S, Cohen G, et al. Staphylococcus aureus ArcR controls expression of the arginine deiminase operon. J Bacteriol. 2007 Aug;189(16):5976–86. doi: 10.1128/JB.00592-07 17557828 PMC1952046

[69] Sause WE, Balasubramanian D, Irnov I, Copin R, Sullivan MJ, Sommerfield A, et al. The purine biosynthesis regulator PurR moonlights as a virulence regulator in Staphylococcus aureus. Proc Natl Acad Sci U S A. 2019 Jul 2;116(27):13563–72. doi: 10.1073/pnas.1904280116 31217288 PMC6613142

[70] Lan L, Cheng A, Dunman PM, Missiakas D, He C. Golden pigment production and virulence gene expression are affected by metabolisms in Staphylococcus aureus. J Bacteriol. 2010 Jun;192(12):3068–77. doi: 10.1128/JB.00928-09 20400547 PMC2901709

[71] Kriegeskorte A, Block D, Drescher M, Windmüller N, Mellmann A, Baum C, et al. Inactivation of thyA in Staphylococcus aureus attenuates virulence and has a strong impact on metabolism and virulence gene expression. mBio. 2014 Jul 29;5(4):e01447–01414. doi: 10.1128/mBio.01447-14 25073642 PMC4128360

[72] Somerville GA, Proctor RA. At the Crossroads of Bacterial Metabolism and Virulence Factor Synthesis in Staphylococci. Microbiol Mol Biol Rev. 2009 Jun;73(2):233–48. doi: 10.1128/MMBR.00005-09 19487727 PMC2698418

[73] Wu S, Zhang J, Peng Q, Liu Y, Lei L, Zhang H. The Role of Staphylococcus aureus YycFG in Gene Regulation, Biofilm Organization and Drug Resistance. Antibiotics (Basel). 2021 Dec 19;10(12):1555. doi: 10.3390/antibiotics10121555 34943766 PMC8698359

[74] Porayath C, Suresh MK, Biswas R, Nair BG, Mishra N, Pal S. Autolysin mediated adherence of Staphylococcus aureus with Fibronectin, Gelatin and Heparin. Int J Biol Macromol. 2018 Apr 15;110:179–84. doi: 10.1016/j.ijbiomac.2018.01.047 29398086 PMC5864509

[75] Shinji H, Yosizawa Y, Tajima A, Iwase T, Sugimoto S, Seki K, et al. Role of fibronectin-binding proteins A and B in in vitro cellular infections and in vivo septic infections by Staphylococcus aureus. Infect Immun. 2011 Jun;79(6):2215–23. doi: 10.1128/IAI.00133-11 21422173 PMC3125839

[76] Lei MG, Gudeta DD, Luong TT, Lee CY. MgrA Negatively Impacts Staphylococcus aureus Invasion by Regulating Capsule and FnbA. Freitag NE, editor. Infect Immun. 2019 Dec;87(12):e00590–19. 31591167 10.1128/IAI.00590-19 PMC6867852

[77] Higgins J, Loughman A, Van Kessel KPM, Van Strijp JAG, Foster TJ. Clumping factor A of *Staphylococcus aureus* inhibits phagocytosis by human polymorphonuclear leucocytes. FEMS Microbiology Letters. 2006 May;258(2):290–6. 16640587 10.1111/j.1574-6968.2006.00229.x

[78] Gajdiss M, Monk IR, Bertsche U, Kienemund J, Funk T, Dietrich A, et al. YycH and YycI Regulate Expression of Staphylococcus aureus Autolysins by Activation of WalRK Phosphorylation. Microorganisms. 2020 Jun 9;8(6):E870. doi: 10.3390/microorganisms8060870 32526915 PMC7355866

[79] Hirschhausen N, Schlesier T, Schmidt MA, Götz F, Peters G, Heilmann C. A novel staphylococcal internalization mechanism involves the major autolysin Atl and heat shock cognate protein Hsc70 as host cell receptor. Cell Microbiol. 2010 Dec;12(12):1746–64. doi: 10.1111/j.1462-5822.2010.01506.x 20642807

[80] Oogai Y, Matsuo M, Hashimoto M, Kato F, Sugai M, Komatsuzawa H. Expression of virulence factors by Staphylococcus aureus grown in serum. Appl Environ Microbiol. 2011 Nov;77(22):8097–105. doi: 10.1128/AEM.05316-11 21926198 PMC3208999

[81] Barua N, Yang Y, Huang L, Ip M. VraSR Regulatory System Contributes to the Virulence of Community-Associated Methicillin-Resistant Staphylococcus aureus (CA-MRSA) in a 3D-Skin Model and Skin Infection of Humanized Mouse Model. Biomedicines. 2021 Dec 24;10(1):35. doi: 10.3390/biomedicines10010035 35052714 PMC8772825

[82] Freeman ZN, Dorus S, Waterfield NR. The KdpD/KdpE two-component system: integrating K+ homeostasis and virulence. PLoS Pathog. 2013 Mar;9(3):e1003201. 23555240 10.1371/journal.ppat.1003201 PMC3610689

[83] Goerke C, Fluckiger U, Steinhuber A, Bisanzio V, Ulrich M, Bischoff M, et al. Role of Staphylococcus aureus global regulators sae and sigmaB in virulence gene expression during device-related infection. Infect Immun. 2005 Jun;73(6):3415–21. doi: 10.1128/IAI.73.6.3415-3421.2005 15908369 PMC1111833

[84] DelMain EA, Moormeier DE, Endres JL, Hodges RE, Sadykov MR, Horswill AR, et al. Stochastic Expression of Sae-Dependent Virulence Genes during Staphylococcus aureus Biofilm Development Is Dependent on SaeS. mBio. 2020 Jan 14;11(1):e03081–19. doi: 10.1128/mBio.03081-19 31937649 PMC6960292

[85] Yang D, Ho YX, Cowell LM, Jilani I, Foster SJ, Prince LR. A Genome-Wide Screen Identifies Factors Involved in S. aureus-Induced Human Neutrophil Cell Death and Pathogenesis. Front Immunol. 2019 Jan 31;10:45. 10.3389/fimmu.2019.00045 PMC6365652 30766531

[86] Xiong YQ, Li Y, Goncheva MI, Elsayed AM, Zhu F, Li L, et al. The Purine Biosynthesis Repressor, PurR, Contributes to Vancomycin Susceptibility of Methicillin-resistant Staphylococcus aureus in Experimental Endocarditis. J Infect Dis. 2024 Jan 31;jiad577. doi: 10.1093/infdis/jiad577 38297970 PMC11175694

[87] Paudel A, Panthee S, Hamamoto H, Grunert T, Sekimizu K. YjbH regulates virulence genes expression and oxidative stress resistance in Staphylococcus aureus. Virulence. 2021 Dec;12(1):470–80. doi: 10.1080/21505594.2021.1875683 33487122 PMC7849776

[88] Herr AB, Thorman AW. Hiding in plain sight: immune evasion by the staphylococcal protein SdrE. Biochemical Journal. 2017 Jun 1;474(11):1803–6. doi: 10.1042/BCJ20170132 28490660

[89] Kim GL, Akoolo L, Parker D. The ClpXP Protease Contributes to Staphylococcus aureus Pneumonia. J Infect Dis. 2020 Sep 14;222(8):1400–4. doi: 10.1093/infdis/jiaa251 32386322 PMC7488198

[90] Connolly J, Boldock E, Prince LR, Renshaw SA, Whyte MK, Foster SJ. Identification of Staphylococcus aureus Factors Required for Pathogenicity and Growth in Human Blood. Infect Immun. 2017;85(11). doi: 10.1128/IAI.00337-17 28808156 PMC5649012

[91] Marroquin S, Gimza B, Tomlinson B, Stein M, Frey A, Keogh RA, et al. MroQ Is a Novel Abi-Domain Protein That Influences Virulence Gene Expression in Staphylococcus aureus via Modulation of Agr Activity. Infect Immun. 2019 Mar;87(5):e00002–19. doi: 10.1128/IAI.00002-19 30833335 PMC6479029

[92] O’Halloran DP, Wynne K, Geoghegan JA. Protein A is released into the Staphylococcus aureus culture supernatant with an unprocessed sorting signal. Infect Immun. 2015 Apr;83(4):1598–609. doi: 10.1128/IAI.03122-14 25644005 PMC4363444

[93] Becker S, Frankel MB, Schneewind O, Missiakas D. Release of protein A from the cell wall of Staphylococcus aureus. Proc Natl Acad Sci U S A. 2014 Jan 28;111(4):1574–9. doi: 10.1073/pnas.1317181111 24434550 PMC3910568

[94] Bayer AS, McNamara P, Yeaman MR, Lucindo N, Jones T, Cheung AL, et al. Transposon Disruption of the Complex I NADH Oxidoreductase Gene *(snoD)* in *Staphylococcus aureus* Is Associated with Reduced Susceptibility to the Microbicidal Activity of Thrombin-Induced Platelet Microbicidal Protein 1. J Bacteriol. 2006 Jan;188(1):211–22. 16352837 10.1128/JB.188.1.211-222.2006 PMC1317573

[95] Streker K, Freiberg C, Labischinski H, Hacker J, Ohlsen K. *Staphylococcus aureus* NfrA (SA0367) Is a Flavin Mononucleotide-Dependent NADPH Oxidase Involved in Oxidative Stress Response. J Bacteriol. 2005 Apr;187(7):2249–56. 15774866 10.1128/JB.187.7.2249-2256.2005 PMC1065224

[96] Loughlin MF, Arandhara V, Okolie C, Aldsworth TG, Jenks PJ. Helicobacter pylori mutants defective in the clpP ATP-dependant protease and the chaperone clpA display reduced macrophage and murine survival. Microb Pathog. 2009 Jan;46(1):53–7. doi: 10.1016/j.micpath.2008.10.004 18992803

[97] Vaish M, Price-Whelan A, Reyes-Robles T, Liu J, Jereen A, Christie S, et al. Roles of Staphylococcus aureus Mnh1 and Mnh2 Antiporters in Salt Tolerance, Alkali Tolerance, and Pathogenesis. J Bacteriol. 2018 Mar 1;200(5). 10.1128/JB.00611-17 PMC5809693 29263099

[98] Casey D, Sleator RD. A genomic analysis of osmotolerance in Staphylococcus aureus. Gene. 2021 Jan;767:145268. doi: 10.1016/j.gene.2020.145268 33157201

[99] Willing S, Dyer E, Schneewind O, Missiakas D. FmhA and FmhC of Staphylococcus aureus incorporate serine residues into peptidoglycan cross-bridges. J Biol Chem. 2020 Sep 25;295(39):13664–76. doi: 10.1074/jbc.RA120.014371 32759309 PMC7521636

[100] Berger-Bächi B, Tschierske M. Role of fem factors in methicillin resistance. Drug Resist Updat. 1998;1(5):325–35. doi: 10.1016/s1368-7646(98)80048-4 17092813

[101] Weidenmaier C, Peschel A. Teichoic acids and related cell-wall glycopolymers in Gram-positive physiology and host interactions. Nat Rev Microbiol. 2008 Apr;6(4):276–87. doi: 10.1038/nrmicro1861 18327271

[102] van Dalen R, Peschel A, van Sorge NM. Wall Teichoic Acid in Staphylococcus aureus Host Interaction. Trends in Microbiology. 2020 Dec;28(12):985–98. doi: 10.1016/j.tim.2020.05.017 32540314

[103] Wang M, Buist G, van Dijl JM. Staphylococcus aureus cell wall maintenance—the multifaceted roles of peptidoglycan hydrolases in bacterial growth, fitness, and virulence. FEMS Microbiol Rev. 2022 Oct 28;46(5):fuac025. doi: 10.1093/femsre/fuac025 35675307 PMC9616470

[104] Sutton JAF, Carnell OT, Lafage L, Gray J, Biboy J, Gibson JF, et al. Staphylococcus aureus cell wall structure and dynamics during host-pathogen interaction. PLoS Pathog. 2021 Mar;17(3):e1009468. doi: 10.1371/journal.ppat.1009468 33788901 PMC8041196

[105] Oliver A, Mena A. Bacterial hypermutation in cystic fibrosis, not only for antibiotic resistance. Clinical Microbiology and Infection. 2010 Jul;16(7):798–808. doi: 10.1111/j.1469-0691.2010.03250.x 20880409

[106] Mclean K, Lee D, Holmes E, Penewit K, Waalkes A, Ren M, et al. Genomic analysis identifies novel Pseudomonas aeruginosa resistance genes under selection during inhaled aztreonam therapy in vivo. Antimicrobial Agents and Chemotherapy. doi: 10.1128/AAC.00866-19 31285231 PMC6709462

[107] Porto PD, Cifani N, Guarnieri S, Di Domenico EG, Mariggiò MA, Spadaro F, et al. Dysfunctional CFTR Alters the Bactericidal Activity of Human Macrophages against Pseudomonas aeruginosa. Nigou J, editor. PLoS ONE. 2011 May 18;6(5):e19970. doi: 10.1371/journal.pone.0019970 21625641 PMC3097223

[108] Shah PL, Mawdsley S, Nash K, Cullinan P, Cole PJ, Wilson R. Determinants of chronic infection with Staphylococcus aureus in patients with bronchiectasis. Eur Respir J. 1999 Dec;14(6):1340–4. doi: 10.1183/09031936.99.14613409 10624764

[109] Clement S, Vaudaux P, Francois P, Schrenzel J, Huggler E, Kampf S, et al. Evidence of an intracellular reservoir in the nasal mucosa of patients with recurrent Staphylococcus aureus rhinosinusitis. J Infect Dis. 2005 Sep 15;192(6):1023–8. doi: 10.1086/432735 16107955

