# Supplementary material for: Clinical and in vitro models identify distinct adaptations enhancing Staphylococcus aureus pathogenesis in human macrophages
Source: PLoS Pathog. 2024 Jul 11;20(7):e1012394. doi: 10.1371/journal.ppat.1012394 (PMC11265673; doi:10.1371/journal.ppat.1012394)
Supplement: S1 Fig — Quantile-quantile plots for in vivo mutations associated with macrophage invasion (blue) and survival (red) phenotypes by various implementations of genome-wide association studies. Association between phenotypes by analysis of (A) reference-free sequence differences (“unitigs”), (B) non-synonymous mutations with gene kernels (“burden testing”), and (C) de novo mutations arising within clonally related patient lineages over time (“first-last comparison”). Significant and non-significant results are shaded differentially. (PDF) [file ppat.1012394.s014.pdf]

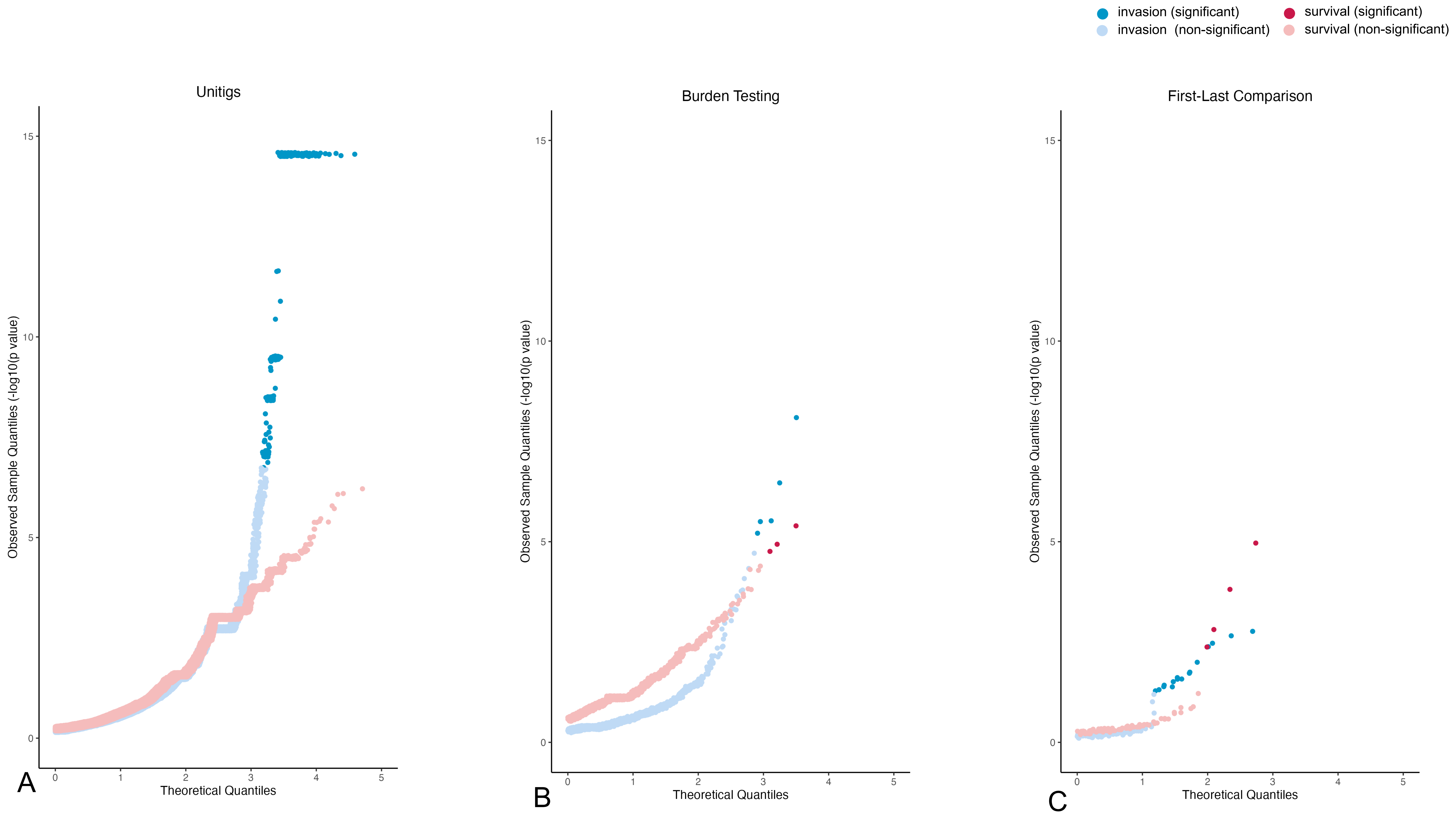

invasion (significant)
invasion (non-significant)
survival (significant)
survival (non-significant)
Unitigs
Burden Testing
First-Last Comparison
Observed Sample Quantiles (-log10(p value)
Theoretical Quantiles
0
5
10
15
0
1
2
3
4
5
A
B
C
