# Supplementary figures and images for: Clinical and in vitro models identify distinct adaptations enhancing Staphylococcus aureus pathogenesis in human macrophages
Source: PLoS Pathog. 2024 Jul 11;20(7):e1012394. doi: 10.1371/journal.ppat.1012394 (PMC11265673; doi:10.1371/journal.ppat.1012394)

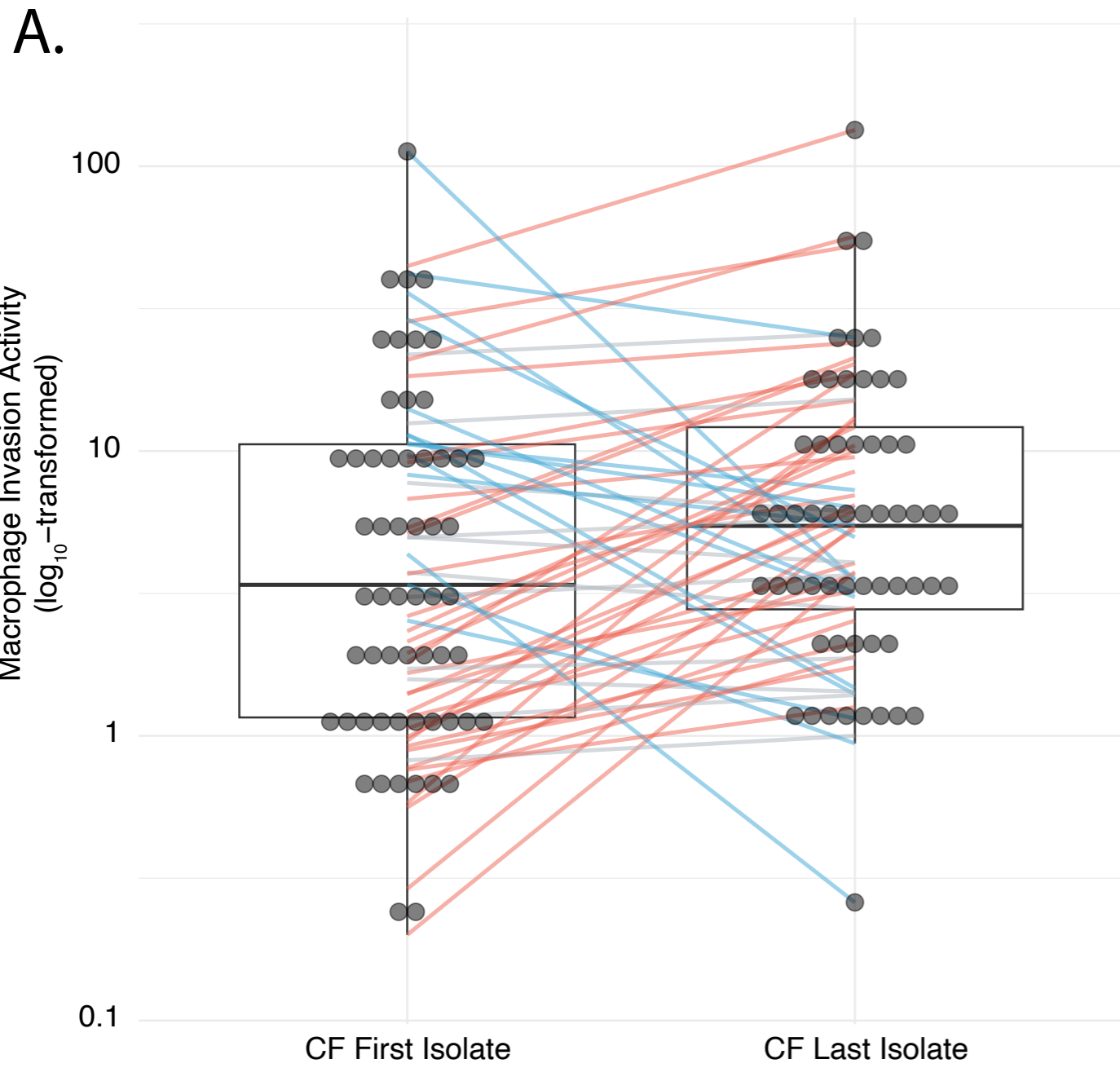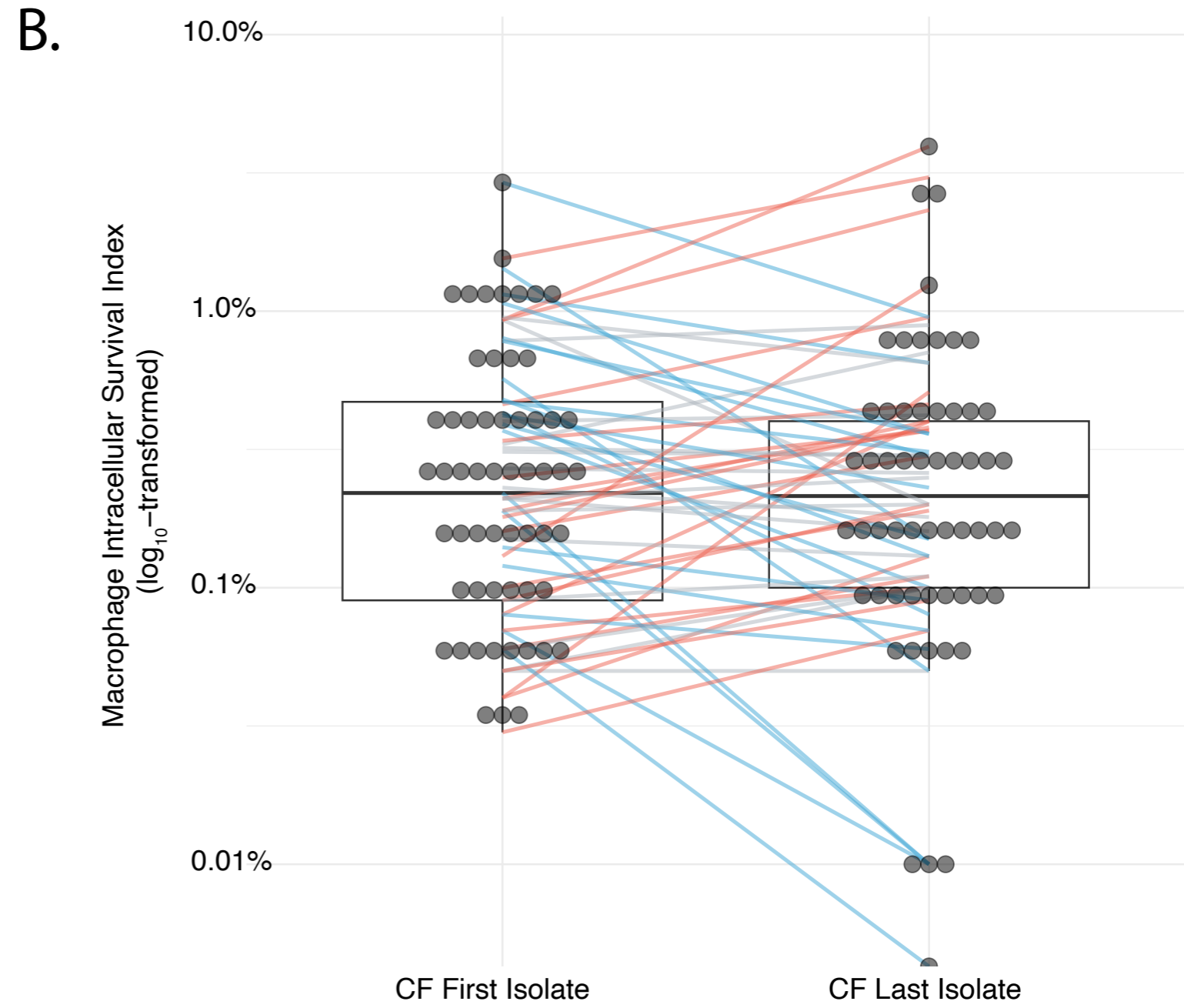

Supplement: S2 Fig — Phenotypes of S. aureus strains isolated from respiratory cultures of individuals with CF at the time of initial isolation during serial surveillance are compared to the last-collected strains from same patient. Colored lines in this connected dot plot indicate the direction and significance of changes in individual first-last isolate pairs (red = significant increase over time, blue = significant decrease over time, grey = no-significant change) for measures of macrophage invasion relative to in-batch testing of the JE2 control strain (A), and macrophage intracellular survival (B). Statistical significance was assessed using paired two-tailed T tests. (PDF) [file ppat.1012394.s015.pdf]
